# EasyCatch, a convenient, sensitive and specific CRISPR detection system for cancer gene mutations

**DOI:** 10.1186/s12943-021-01456-x

**Published:** 2021-12-02

**Authors:** Yin Liu, Yanling Chen, Lu Dang, Yixin Liu, Shisheng Huang, Sanyun Wu, Peixiang Ma, Hongqiang Jiang, Yi Li, Yunbao Pan, Yongchang Wei, Xiaodong Ma, Ming Liu, Quanjiang Ji, Tian Chi, Xingxu Huang, Xinjie Wang, Fuling Zhou

**Affiliations:** 1 Department of Hematology, Zhongnan Hospital of Wuhan University, No.169 Donghu Road, Wuhan, 430072 China; 2 Department of Reproductive Medicine, Third Affiliated Hospital of Guangzhou Medical University, Guangzhou, 510150 China; 3 Department of Radiation and Medical Oncology, Zhongnan Hospital of Wuhan University, No.169 Donghu Road, Wuhan, 430072 China; 4 Gene Editing Center, School of Life Science and Technology, ShanghaiTech University, 100 Haike Rd., Pudong New Area, Shanghai, 201210 China; 5 Shanghai Institute for Advanced Immunochemical Studies, ShanghaiTech University, Shanghai, 201210 China; 6 Department of Laboratory Medicine, Zhongnan Hospital of Wuhan University, Wuhan University, No.169 Donghu Road, Wuhan, 430072 China; 7 Institute for Brain Research and Rehabilitation, Guangdong Key Laboratory of Mental Health and Cognitive Science, Center for Studies of Psychological Application, South China Normal University, Guangzhou, 510631 China; 8 State Key Laboratory of Respiratory Disease/National Clinical Research Center for Respiratory Disease/National Center for Respiratory Medicine/Guangzhou Institute of Respiratory Health/The First Affiliated Hospital of Guangzhou Medical University, Guangzhou, 510120 China; 9 School of Physical Science and Technology, ShanghaiTech University, 100 Haike Rd., Pudong New Area, Shanghai, 201210 China; 10 Shenzhen Branch, Guangdong Laboratory of Lingnan Modern Agriculture, Genome Analysis Laboratory of the Ministry of Agriculture and Rural Affairs, Agricultural Genomics Institute at Shenzhen, Chinese Academy of Agricultural Sciences, Shenzhen, China

**Keywords:** CRISPR detection, cancer mutation, Drug resistance, Leukemia, SNP detection

## Main text

Convenient, sensitive and specific detection of rare genetic variants and mutations is essential for early cancer diagnosis and precision medicine [1], but tools that are simultaneously endowed with all these attributes remain elusive despite years of intense quest. Recently, Clustered Regularly Interspaced Short Palindromic Repeats (CRISPR) associated (Cas) proteins have shown great potential for rapid and sensitive nucleic acid detection [2–5]. Guided by a CRISPR-derived RNA (crRNA) complementary to the target sequence, the CRISPR/Cas complex can recognize and cleave the target nucleic acid with single-base resolution specificity [6]. Cas12a is one of the most commonly used CRISPR/Cas proteins for DNA detection. Upon recognition and cleavage of target double-strand DNA, the collateral cleavage activity of Cas12a is activated, resulting in cleavage of nearby single-strand DNA (including fluorescence reporters) in a non-specific manner [7], and this “collateral cleavage” has been widely exploited for sensitive and specific detection of target sequences [8, 9]. However, for detection of rare mutations in cancers, the majority of the DNA are wild-type (WT) sequences, significantly hampering the analysis. We have now solved the problem by simply including restriction digestion in the detection system, making our method (EasyCatch, for Excision-amplification-synchronous Cas12a-targeted checkout) the first capable of convenient, specific, sensitive (0.001%), and rapid (< 1 h) detection of mutations in cancer samples.

### Development of EasyCatch

**Fig. 1:**
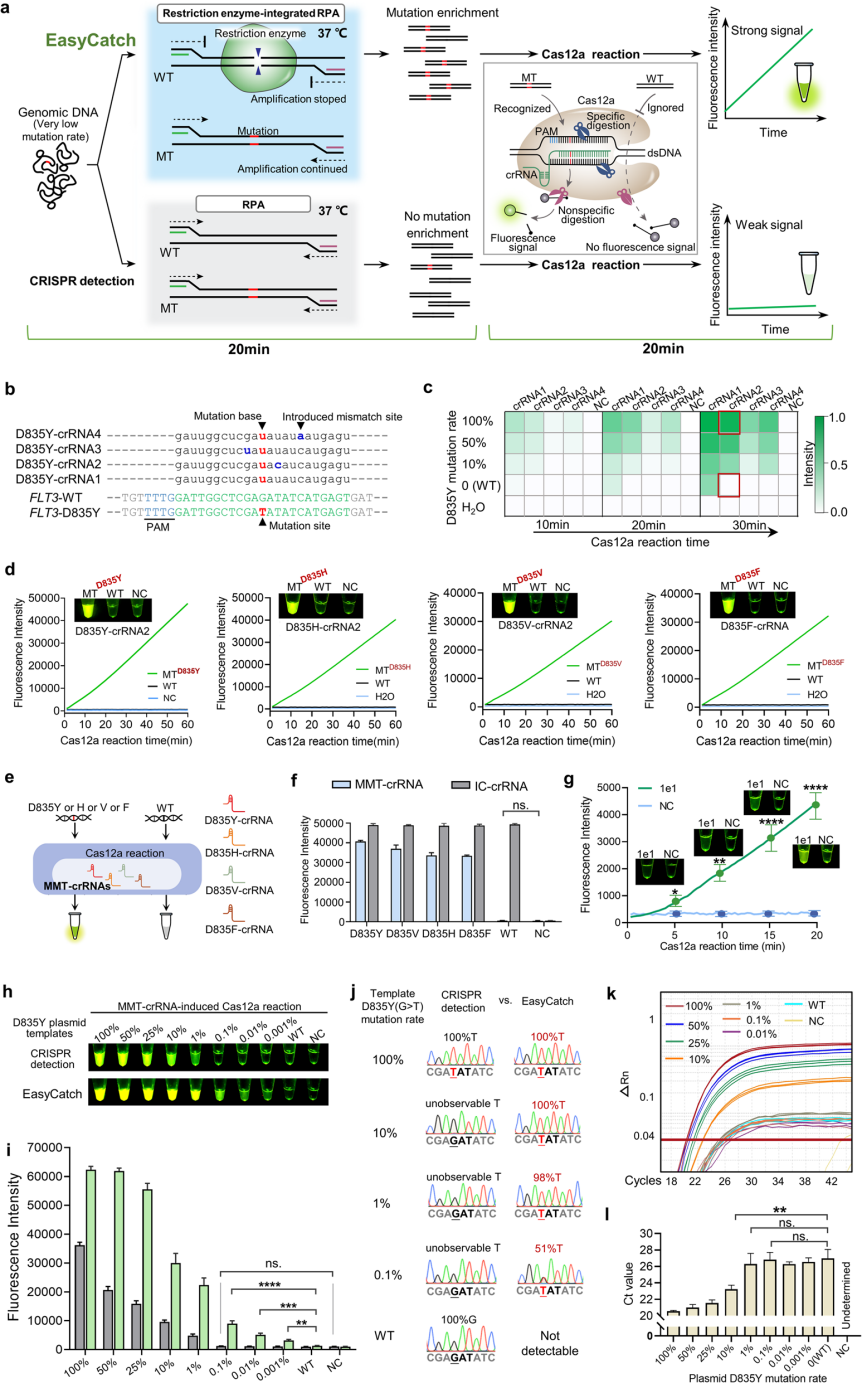
Development and validation of EasyCatch system for specific and sensitive mutation detection. **a** Schemes of EasyCatch compared with CRISPR detection. **b** Sequences of D835Y-crRNAs, and *FLT3*-WT and D835Y gene region. Base G mutates to T in D835Y. **c** Fluorescence heatmap of different D835Y-crRNA-induced Cas12a reactions detecting 1e10 copies of PCR fragments with different D835Y mutation rates (100, 50, 10%, and 0), Cas12a reactions for 10, 20 and 30 min were recorded. **d** Specificity assay of D835Y-crRNA2, D835H-crRNA, D835V-crRNA, and D835F-crRNA. Time-course of fluorescence intensity and naked-eye observation after 60 min of Cas12a reaction are shown. **e** Schematic diagram of MMT-crRNAs-guided Cas12a reaction to identify D835Y/H/V/F mutations from WT background. **f** Specificity assay of MMT-crRNAs using 1e10 copies of D835Y/H/V/F and WT fragments. Fluorescence intensity after 60 min of Cas12a reaction are shown. IC, inner control. **g** Time-course analysis of the detection of 1e1 D835Y plasmid templates by RPA combined with MMT-crRNAs induced Cas12a reaction. **h, i** Sensitivity comparison of EasyCatch and CRISPR detection in detecting 1e6 copies of plasmid templates with gradient D835Y mutation rates. **j** FGS results of the amplified products in EasyCatch and CRISPR detection. The D835Y mutation rates were quantified using the online tool EditR (https://moriaritylab.shinyapps.io/editr_v10/). **k** The design and amplification plot of D835Y-probe-mediated qPCR in detecting 1e6 copies of plasmid templates with gradient D835Y mutation rates. **l** Ct value comparison of different qPCR samples

For traditional CRISPR detection, when the target mutation sequence in the samples constitutes only a minority of total DNA, the detection becomes difficult due to the interference by the non-target WT sequences, even after target enrichment by recombinase polymerase amplification (RPA, in 20 min at 37 ~ 42 °C) (Fig. 1a) [10]. We hypothesized that by adding a restriction enzyme recognizing the WT sequence to the RPA system, we can destroy the interfering sequences, and thereby inhibit the amplification of the WT sequences while facilitate the amplification of mutant templates, thus increasing the detection sensitivity (Fig. 1a). This strategy is the basis of EasyCatch.

For proof-of-concept demonstration, we used EasyCatch to detect the drug-resistant *FLT3* gene D835Y/H/V/F mutations in acute myeloid leukemia (AML) [11]. Since EcoRV restriction enzyme can recognize and digest WT D835 sequence (−GATATC-), but not the D835 mutant sequences (Fig. S1a). The feasibility of EcoRV digestion in the RPA reaction was validated using PCR fragments, wherein the fluorescence signal of WT D835 fragments was eliminated by EcoRV digestion, while that of the D835Y/H/V/F fragments was enhanced (Fig. S1b-d).

In a Cas12a reaction, a mutation-specific crRNA is required to avoid the cross-reactivity against WT. To screen for the optimal crRNA for detecting *FLT3*-D835Y, we designed four crRNAs, with *FLT3*-D835Y-crRNA1 perfectly matching the mutant sequence and *FLT3*-D835Y-crRNA2 ~ 4 bearing various mismatches (Fig. 1b). We compared the sensitivity and specificity of these four crRNAs in detecting D835Y in PCR fragments (1e10 copies) comprising the D835Y and WT alleles. After 30 min of the Cas12a reaction, all the 4 crRNAs, particularly crRNA1 and crRNA2, could detect the sample with 100% D835Y, while crRNA1 but not crRNA2 produced a strong signal even for WT sample (Fig. 1c; S2). Thereby crRNA2 is the optimal crRNA for D835Y detection based on its excellent sensitivity and specificity. Optimal crRNAs for D835H/V/F and WT were similarly identified, which are D835H-crRNA2, D835V-crRNA2, D835F-crRNA, WT-crRNA2, respectively (Fig. 1d; S3–6), and an inner control (IC)-crRNA was also designed to target a non-mutation sequence near the mutation site. To simplify the diagnosis of the four mutations, we pooled the D835Y/H/V/F crRNAs (MMT-crRNAs) and the 4 mutant fragments into a single reaction, resulting in a strong fluorescence signal, whereas the WT sample did not show any signal, as expected (Fig. 1e, f; S7).

Next, we screened 9 pairs of RPA primers to improve EasyCatch sensitivity (Fig. S8). Using the best primers and MMT-crRNAs, as low as 10 copies of D835Y plasmid templates could be detected only after 20 min of RPA and 20 min of Cas12a reaction (Fig. 1g). Therefore, we chose this primer pair and 20 min as the Cas12a reaction time in the subsequent experiments.

### EasyCatch achieves a 0.001% sensitivity in *FLT3*-D835Y detection

With the optimized crRNAs and primers, we set out to determine the detection limit of EasyCatch for *FLT3*-D835Y. We first quantified the effect of EcoRV on WT sequence, finding that EcoRV could completely inhibit the amplification of up to 1e6 copies of WT templates but spared the mutant target as expected (Fig. S9, 10). We then mixed the mutant and WT templates at various ratios, with *FLT3*-D835Y comprised 100, 50, 25, 10, 1, 0.1, 0.01, and 0.001% of the total templates, and used 1e6 copies of the templates as the input for EasyCatch, finding the detection limit being 0.001% (i.e., 10 copies of D835Y template amid 999,990 copies of WT template). In contrast, in the absence of EcoRV, namely CRISPR detection, the detection limit was 1000x lower (1%) (Fig. 1h, i; S11). Finally, FGS directly demonstrated that EcoRV markedly enriched the mutant allele in the RPA mixture from 10, 1 and 0.1% to 100, 98 and 51%, respectively (Fig. 1j).

We next compared EasyCatch with the commonly used qPCR-based detection method. We designed and tested two D835Y-specific TaqMan probes (Fig. S12), and then chose the more specific one (probe 1) for the comparison (Fig. S13, 14). The qPCR results showed that the amplification curves and Ct values of 1, 0.1, 0.01% mutant samples cannot be distinguished from that of the WT sample (Fig. 1k, l), indicating its sensitivity of only 1% ~ 10%. Together, these results suggested that EasyCatch is much more sensitive than TaqMan qPCR in detecting *FLT3*-D835Y mutation.

### EasyCatch accurately detected *FLT3*-D835Y/V/H/F mutations in clinical samples

**Fig. 2:**
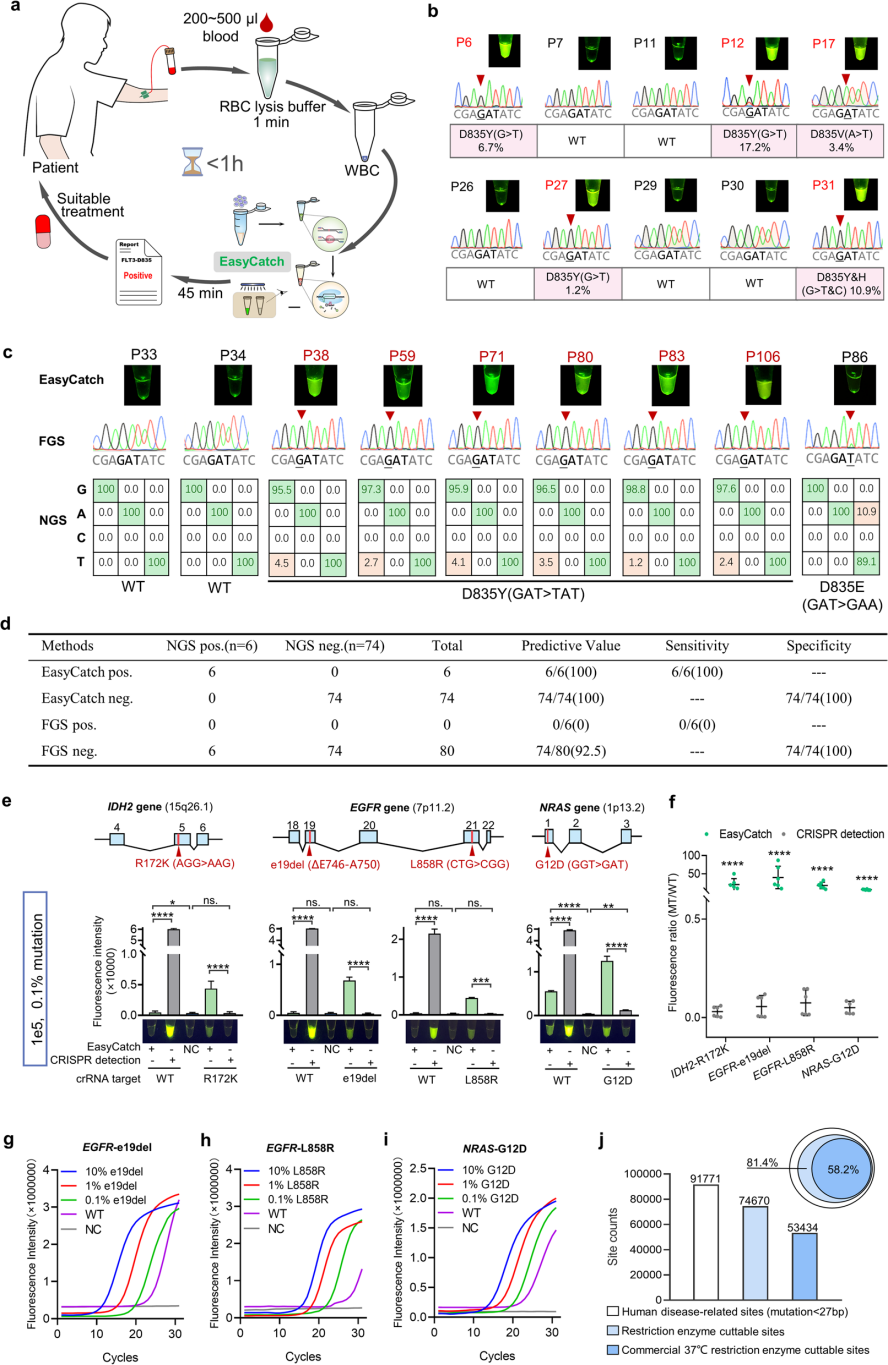
EasyCatch is applicable to clinical samples and other cancer mutations. **a** Schematic diagram of the whole mutation diagnosis. **b** EasyCatch and FGS results of 32 AML samples with known *FLT3*-D835 mutation status, 10/32 cases are shown. D835Y/V/H/F-positive patients are marked by red IDs, and red triangles indicate mutant bases. **c** EasyCatch, FGS, and NGS results of 80 AML samples with unknown *FLT3*-D835 mutation status, 9/80 cases are shown. WT and mutated bases in NGS are marked by green and red, respectively. **d** Statistical table of the sensitivity and specificity of EasyCatch compared with FGS using NGS as a standard reference. **e** Sensitivity comparison between EasyCatch and CRISPR detection in the detection of *IDH2*-R172K, *EGFR*-e19del and L858R, and *NRAS*-G12D mutations. Genomic locations of these mutations were shown above, wherein exons and mutation sites were colored in blue and red, respectively. The tested samples were 1e5 copies of plasmid templates with a mutation rate of 0.1%. Each amplified product was detected by both WT-crRNA and mutation-crRNA induced Cas12a reaction. Fluorescence intensity and naked eye results were both recorded. **f** Statistic analysis of the MT/WT fluorescence ratio in EasyCatch and CRISPR detection. The results of 1 and 0.1% mutated samples were counted together. **g**, **h**, **i** The qPCR assay for *EGFR*-e19del, L858R and *NRAS*-G12D detection, respectively. The qPCRs were performed on 10, 1 and 0.1% mutated templates. A 100% WT template and ddH_2_O (NC) served as control. **j** The statistical chart of restriction enzyme cuttable human disease-related genomic sites (mutation < 27 bp, which is the detection length of crRNA), wherein commercial available 37 °C restriction enzyme cuttable sites can be potentially detected by EasyCatch. The inclusion relation is shown in the upper-right corner

With these, AML patient samples were used for EasyCatch detection of D835Y/V/H/F mutations. Briefly, 200 ~ 500 μl of blood was incubated with red blood cell (RBC) lysis buffer for 1 min, and the cell precipitate was obtained by 1 min of centrifugation. Next, genomic DNA was released by a nucleic acid releaser under 95 °C for 3 min and then treated by the EasyCatch assay (Fig. S15). From drawing blood to making treatment decisions, the whole process can be completed within 1 h (Fig. 2a). We then simultaneously applied EasyCatch and first-generation sequencing (FGS) to detect 32 AML samples whose *FLT3* gene mutation status had been analyzed by next-generation sequencing (NGS) previously, wherein P6, P12, P17, P27, and P31 carried drug-resistant D835Y/V/H/F mutations (Fig. 2b; S16, 17). The results showed that EasyCatch successfully identified all five mutant samples; in contrast, only two samples with relatively high mutation rates, P12 of 17.2% and P31 of 10.9%, were identified by FGS (Fig. 2b). Thus, EasyCatch outperforms FGS for clinical sample analysis. More importantly, the EasyCatch method is simple and economical, and only needs a mini centrifuge, a 20 μl pipette and tips, a heat blocker, and a blue lamp with a 485 nm wavelength (Fig. S18).

We next benchmarked EasyCatch against the commonly used FGS for detecting *FLT3*-D835 mutations in 80 AML patients (P33 -P112) with unknown *FLT3*-D835 mutation status, with NGS as the gold standard. EasyCatch, but not FGS, was able to detect D835Y in P38, P59, P71, P80, P83, and P106 (Fig. 2c). NGS confirmed that all the six samples harbored the D835Y mutation (at 4.5, 2.7, 4.1, 3.5, 1.2 and 2.4%, respectively) (Fig. S19, 20). Notably, NGS showed that P86 carried 10.9% non-drug-resistant D835E (GAT>GAA) mutation. As this mutation did not produce a signal in EasyCatch, further confirming the high specificity of this method (Fig. 2c). The statistical analysis of the 80 cases further showed that EasyCatch is much more sensitive than FGS (100% vs. 0%) (Fig. 2d).

### EasyCatch is applicable to other cancer mutations

To verify the versatility of EasyCatch, we applied it to detect 4 mutations at 3 other genes (*IDH2*, *EGFR* and *NRAS*). *IDH2* R172K is a hotspot mutation in glioma and leukemia, and of prognostic and therapeutic value [12, 13]. *EGFR* e19del and L858R are the two main mutations sensitive to *EGFR*-TKIs, thus of great therapeutic value for patients with lung cancer [14]. At the same time, the *NRAS* G12D is a driver mutation in leukemia and colorectal cancer [15]. All four mutations are important testing items in the clinic. We first compared EasyCatch with the CRISPR detection method to detect 1e5 copies of plasmid templates of 1 and 0.1% mutation rate. The results showed that the WT fluorescence signals were strong while the signals of all the four mutations were weak in CRISPR detection. However, the WT signals were almost invisible while the mutation signals were significantly increased in EasyCatch (Fig. 2e; S21a). The FGS results of the amplified products also confirmed the excellent mutation enrichment effect of EasyCatch (Fig. S21b). Further analysis indicated that the MT/WT fluorescence ratios in EasyCatch were hundreds of times higher than that of CRISPR detection (Fig. 2f). More samples with decreasing frequencies (100, 50, 25, 10, 1, 0.1%, and 0) of these mutations were also tested, with consistent results (Fig. S22–25).

We also detected *EGFR*-e19del, *EGFR*-L858R, and *NRAS*-G12D mutations using commercial kits based on fluorescence qPCR. The tested samples were 1e5 copies of plasmid templates with a mutation rate of 10, 1, 0.1, and 0% (WT), respectively. The results of all three sites showed that the amplification curves of different samples were gradually shifted to right, consistent with the decreased mutation rate. However, we noticed strong fluorescence signals in WT samples (Fig. 2g-i; S26–28). In our EasyCatch detection, the WT signal was completely inhibited by restriction digestion and mutation-specific crRNA, ensuring the reliability of the results.

Finally, we predicted the scope of application of EasyCatch. Total 91,771 human disease-related sites have been documented in the ClinVar database (www.ncbi.nlm.nih.gov/clinvar), and more than 63,000 restriction enzymes reported in the REBASE database (http://rebase.neb.com/rebase/azlist.re2.html), 485 of them commercially available and functioning at 37 °C. We found that 53,434 (58.2%) sites can be cut by the 485 enzymes, indicating that the EasyCatch system can potentially diagnose ~ 60% of human disease-related mutations (Fig. 2j). In addition to restriction enzymes, PAM requirement also imposes restriction on the target range of EasyCatch, but this can be alleviated using Cas12a variants (Table S5), and primer-introduced PAM (Fig. S29). Thus, EasyCatch is a versatile method for detecting gene mutations.

## Conclusions

We have adapted the Cas12a-based DNA detection platform for evaluating rare cancer-related mutations by establishing EasyCatch assay with the sensitivity of 0.001%, which can be completed (from sample preparation to data output) within an hour using only simple instruments and operations. Our study established EasyCatch as the first mutation detection method that is not only superbly sensitive and specific, but also extremely fast, simple, and convenient, showing its versatility in point-of-care cancer diagnosis and precision medicine.

## Supplementary Information

**Additional file 1.** Methods.

**Additional file 2: Supplementary Fig. 1** Validation of the activity and specificity of EcoRV in RPA system. **Supplementary Fig. 2** Specificity assay of *FLT3*-D835Y-crRNAs. **Supplementary Fig. 3** Specificity assay of *FLT3*-D835H-crRNAs. **Supplementary Fig. 4** Specificity assay of *FLT3*-D835V-crRNAs. **Supplementary Fig. 5** Specificity assay of *FLT3*-D835F-crRNAs. **Supplementary Fig. 6** Specificity assay of *FLT3*-D835WT-crRNAs. **Supplementary Fig. 7** Specificity assay of MMT-crRNAs. **Supplementary Fig. 8** RPA primer screen for highly sensitive EasyCatch detection. **Supplementary Fig. 9** WT inhibition assay by RPA with or without EcoRV. **Supplementary Fig. 10** Detection of 1e6 ~ 1e1 copies of D835Y and WT plasmids using RPA with or without EcoRV. **Supplementary Fig. 11** Inhibition of WT amplification by EasyCatch. **Supplementary Fig. 12** Design of TaqMan qPCR for the detection of *FLT3*-D835Y. **Supplementary Fig. 13** The amplification plot of D835Y-probe 1-involved qPCR in detecting 1e5 copies of plasmid templates with gradient D835Y mutation rates. **Supplementary Fig. 14** The amplification plot of D835Y-probe 2-involved qPCR in detecting 1e5 copies of plasmid templates with gradient D835Y mutation rates.

**Additional file 3: Supplementary Fig. 15** Exploration of the fastest blood processing time before EasyCatch. **Supplementary Fig. 16** Classification information and NGS results of the *FLT3*-D835 mutation status of 32 AML samples. **Supplementary Fig. 17** EasyCatch and FGS results of the 32 AML samples in the detection of *FLT3*-D835Y/V/H/F nutations. **Supplementary Fig. 18** Equipment needed in EasyCatch assay. **Supplementary Fig. 19** EasyCatch results of 80 AML patient samples read by naked eyes. **Supplementary Fig. 20** EasyCatch, FGS and NGS results of 80 AML patient blood samples. **Supplementary Fig. 21** Broad application of EasyCatch in cancer mutation diagnosis. **Supplementary Fig. 22** Comparison of CRISPR detection and EasyCatch on *IDH2*-R172K mutation. **Supplementary Fig. 23** Comparison of CRISPR detection and EasyCatch on *EGFR*-e19del mutation. **Supplementary Fig. 24** Comparison of CRISPR detection and EasyCatch on *EGFR*-L858R mutation. **Supplementary Fig. 25** Comparison of CRISPR detection and EasyCatch on *NRAS*-G12D mutation. **Supplementary Fig. 26** The amplification plot of fluorescence qPCR detection of *EGFR* gene e19del mutation using a commercial kit. **Supplementary Fig. 27** The amplification plot of fluorescence qPCR detection of *EGFR* gene L858R mutation using a commercial kit. **Supplementary Fig. 28** The amplification plot of fluorescence qPCR detection of *EGFR* gene L858R mutation using a commercial kit. **Supplementary Fig. 29** The RPA primer design for *IDH2*-R172K mutation detection.

**Additional file 4: Supplementary Table 1** PCR and RPA primer sequences. **Supplementary Table 2** crRNA sequences. **Supplementary Table 3** Next-generation sequencing primer sequences. **Supplementary Table 4** Primers and probes of Taqman qPCR. **Supplementary Table 5** Cas12a nuclease natural and engineered variants.

## Data Availability

The data supporting the conclusions of this article have been given in this article and its additional files, and can also be requested from the corresponding authors.

## Abbreviations

CRISPR: Clustered Regularly Interspaced Short Palindromic Repeats
Cas: CRISPR associated proteins
AML: Acute myeloid leukemia
*FLT3*: FMS-like tyrosine kinase 3
PCR: Polymerase chain reaction
WT: Wild-type
crRNA: CRISPR RNA
RPA: Recombinase polymerase amplification
RBC: Red blood cell
WBC: White blood cell
FGS: First-generation sequencing
NGS: Next-generation sequencing
