# Supplementary material for: EasyCatch, a convenient, sensitive and specific CRISPR detection system for cancer gene mutations
Source: Mol Cancer. 2021 Dec 2;20:157. doi: 10.1186/s12943-021-01456-x (PMC8638196; doi:10.1186/s12943-021-01456-x)
Supplement: Supplementary file 1 — Additional file 1. Methods. [file 12943_2021_1456_MOESM1_ESM.docx]

**Methods**

**Patient samples**

A total of 112 acute myeloid leukemia (AML) patient samples were collected from the Hematology Department in Zhongnan Hospital of Wuhan University under an approved Institutional Review Board protocol. For genomic DNA extraction, 200 ~ 500 µl peripheral blood or bone marrow blood samples were mixed upside down four times with the red cell lysing reagent (Biosharp, Hefei, China). Then, 1 min of minicentrifuge was used to separate lysed red blood cells (RBCs) and unlysed white blood cells (WBCs). Finally, the precipitated WBCs were split by a 100 µl nucleic acid releaser (Suzhou GenDx Biotech, China) at 95 °C for 3 min to release genomic DNA. Two microliters of the treated sample were used for the subsequent EasyCatch assay.

**Plasmid construction**

A 675 bp DNA fragment covering the WT FMS-like tyrosine kinase 3 (*FLT3*)-D835 site was PCR amplified with primers P1 and P2 from a WT patient's genomic DNA, then fused and cloned into the pGem-T vector (Takara, China). After that, the recombinant plasmids were transformed into *E. coli* DH5α, extracted with an AxyPrep Plasmid Miniprep Kit (Axygen, CA, USA), and quantified by a Nanodrop2000 (Thermo Fisher Scientific, MA, USA). For the construction of *FLT3*-D835Y (c.2503G>T), D835H (c.2503G>C), and D835V (c.2504A>T) plasmids, primers P3 and P4 carrying the D835Y mutation, primers P5 and P6 carrying the D835H mutation, primers P7 and P8 carrying the D835V mutation, and primers P9 and P10 carrying the D835F mutation were used to amplify the wild-type Tvec-*FLT3*-D835 plasmid. Then, the amplified fragments were fused and cloned into the pGem-T vector (Takara, China). In CRISPR RNA (crRNA) screening, a 351 bp DNA fragment harboring the D835 region was amplified with primers P11 and P12 from the above recombinant plasmids, then purified and quantified as mentioned above. The plasmid templates of *IDH2*-R172K, *EGFR*-L858R, *NRAS*-G12D, and their WT forms were constructed in the same way. And the plasmid templates of EGFR-e19del (E746-A750 deletion) and its corresponding WT form were directly synthesized by GenScript (Nanjing, China). The nucleotide sequences of all primers are listed in Supplementary Table S1.

**CRISPR reaction system**

The Cas12a-based detection was performed according to a previous description with modiﬁcations [1]. Briefly, crRNAs were designed according to the target sequences and synthesized by GenScript (Nanjing, China). The nucleotide sequences of all crRNAs are listed in Supplementary Table S2. Cas12a protein was expressed and purified as described previously [2]. The 20 µl reaction system included Cas12a (200 ng/µl, 1 µl), crRNA (100 nM, 1 µl), 10 × NEBuffer 3.1 (2 µl, NEB, MA, USA), RNase inhibitor (1 µl, Novoprotein, China), ssDNA-FQ reporter (25 µM, 1 µl, Genewiz, NJ, USA), an appropriate amount of PCR product or 5 µl Recombinase Polymerase Amplification (RPA) product to test, and supplementary ddH_2_O. After sufficient mixing on the vortex shaker, the mixture was incubated at 37 ℃ for 20 min, and then the green fluorescence signal was visualized under a 485 nm blue lamp (Sangon, Shanghai, China). Fluorescence kinetics were monitored using a monochromator with excitation at 485 nm and emission at 520 nm.

**Recombinase polymerase amplification**

Isothermal amplification of plasmids or patient genomic DNA was conducted by the GenDx ERA Kit (Suzhou GenDx Biotech, China). For CRISPR detection, the 50 µl RPA reaction system included 20 µl reaction buffer, 11 µl ERA basic buffer, 2.5 µl forward primer (10 nM), 2.5 µl reverse primer (10 nM), 2 µl DNA template, 2 µl activator, and supplementary ddH_2_O. For the EasyCatch assay, an additional 2 µl restriction enzyme was mixed into the above RPA system. Fastcut-EcoRV (Monad, Suzhou, China), FastDigest-BseLI, FastDigest-SaqAI, FastDigest-MscI, and FastDigest-BveI (Thermo Fisher Scientific, MA, USA) were used in EasyCatch detection of *FLT3*-D835Y, *IDH2*-R172K, *EGFR*-e19del, *EGFR*-L858R, and *NRAS*-G12D, respectively. Then, the mixture was incubated at 37 ℃ for 20 min. After RPA, 5 µl of the amplification product was transferred to the crRNA-guided Cas12a reaction. The primers for RPA are listed in Supplementary Table S1.

**First-generation sequencing and next-generation sequencing**

For FGS, 25 µl PCR products or 25 µl RPA products were purified using an AxyPrep PCR Clean-up Kit (Axygen, CA, USA) and quantified by a Nanodrop2000 (Thermo Fisher Scientific, MA, USA). For each sample, approximately 300 ng amplified DNA fragments were sent to FGS by Tsingke (Beijing, China). For NGS, different barcoded primers were used to amplify the *FLT3*-D835 region of different samples. The PCR products were purified and mixed equally for NGS by the Illumina NextSeq 500 (2 × 150) platform at the CAS-MPG Partner Institute for Computational Biology Omics Core, Shanghai, China. The primers for NGS are listed in Supplementary Table S3.

**TaqMan qPCR**

TaqMan qPCR probes and primers were designed and synthesized by Tsingke (Beijing, China). The probes were the complementary sequence to the FLT3-D835Y template with 5′ reporter dye FAM and 3′ MGB. The 20 µl qPCR system included 2 × Taq Pro HS Universal Probe Master Mix (10 µl, Vazyme, Nanjing, China), qPCR-F (10 µM × 0.4 µl), qPCR-R (10 µM × 0.4 µl), TaqMan probe (10 µM × 0.2 µl), Template DNA (1 µl), and ddH_2_O (8 µl). PCR cycling conditions were 95 ºC for 30 s and 45 cycles of 95 ºC for 10 s and 60 ºC for 30 s. The sequences of qPCR primers and probes are listed in Table S4. Commercial kits performed the qPCR detection of *EGFR*-e19del, *EGFR*-L858R and *NRAS*-G12D mutations.

**Application scope analysis**

A total of 91,771 human disease-related sites (mutation < 27 bp, which is the detection length of crRNA) were downloaded from the ClinVar database ([www.ncbi.nlm.nih.gov/clinvar](http://www.ncbi.nlm.nih.gov/clinvar)). The digestion spectrum of 63,426 restriction enzymes were downloaded from the REBASE database (<http://rebase.neb.com/rebase/azlist.re2.html>). After removing enzymes with simple recognition sequences such as CC, the 91,771 human disease-related sites were analyzed with the recognition sequences of these restriction enzymes to predict WT-cuttable & MT-uncuttable sites.

**Statistical analysis**

All experiments were repeated three times. Statistical analyses were carried out with GraphPad Prism 8.0. Unpaired two-tailed Student's t-test was used for comparison between two groups. Quantitative data are expressed as mean value ± standard error. *P < 0.05, **P < 0.01, ***P < 0.001, ****P < 0.0001; ns., no significance.

**References**

1. Wang X, Ji P, Fan H, Dang L, Wan W, Liu S, et al. CRISPR/Cas12a technology combined with immunochromatographic strips for portable detection of African swine fever virus. Commun Biol. 2020;3(1)**:**62. <https://doi.org/10.1038/s42003-020-0796-5>

2. Creutzburg SCA, Wu WY, Mohanraju P, Swartjes T, Alkan F, Gorodkin J, Staals RHJ, van der Oost J. Good guide, bad guide: spacer sequence-dependent cleavage efficiency of Cas12a. Nucleic Acids Res. 2020;48(6)**:**3228-3243. <https://doi.org/10.1093/nar/gkz1240>
