# Supplementary material for: EasyCatch, a convenient, sensitive and specific CRISPR detection system for cancer gene mutations
Source: Mol Cancer. 2021 Dec 2;20:157. doi: 10.1186/s12943-021-01456-x (PMC8638196; doi:10.1186/s12943-021-01456-x)
Supplement: Supplementary file 2 — Additional file 2: Supplementary Fig. 1 Validation of the activity and specificity of EcoRV in RPA system. Supplementary Fig. 2 Specificity assay of FLT3-D835Y-crRNAs. Supplementary Fig. 3 Specificity assay of FLT3-D835H-crRNAs. Supplementary Fig. 4 Specificity assay of FLT3-D835V-crRNAs. Supplementary Fig. 5 Specificity assay of FLT3-D835F-crRNAs. Supplementary Fig. 6 Specificity assay of FLT3-D835WT-crRNAs. Supplementary Fig. 7 Specificity assay of MMT-crRNAs. Supplementary Fig. 8 RPA primer screen for highly sensitive EasyCatch detection. Supplementary Fig. 9 WT inhibition assay by RPA with or without EcoRV. Supplementary Fig. 10 Detection of 1e6 ~ 1e1 copies of D835Y and WT plasmids using RPA with or without EcoRV. Supplementary Fig. 11 Inhibition of WT amplification by EasyCatch. Supplementary Fig. 12 Design of TaqMan qPCR for the detection of FLT3-D835Y. Supplementary Fig. 13 The amplification plot of D835Y-probe 1-involved qPCR in detecting 1e5 copies of plasmid templates with gradient D835Y mutation rates. Supplementary Fig. 14 The amplification plot of D835Y-probe 2-involved qPCR in detecting 1e5 copies of plasmid templates with gradient D835Y mutation rates. [file 12943_2021_1456_MOESM2_ESM.pdf]

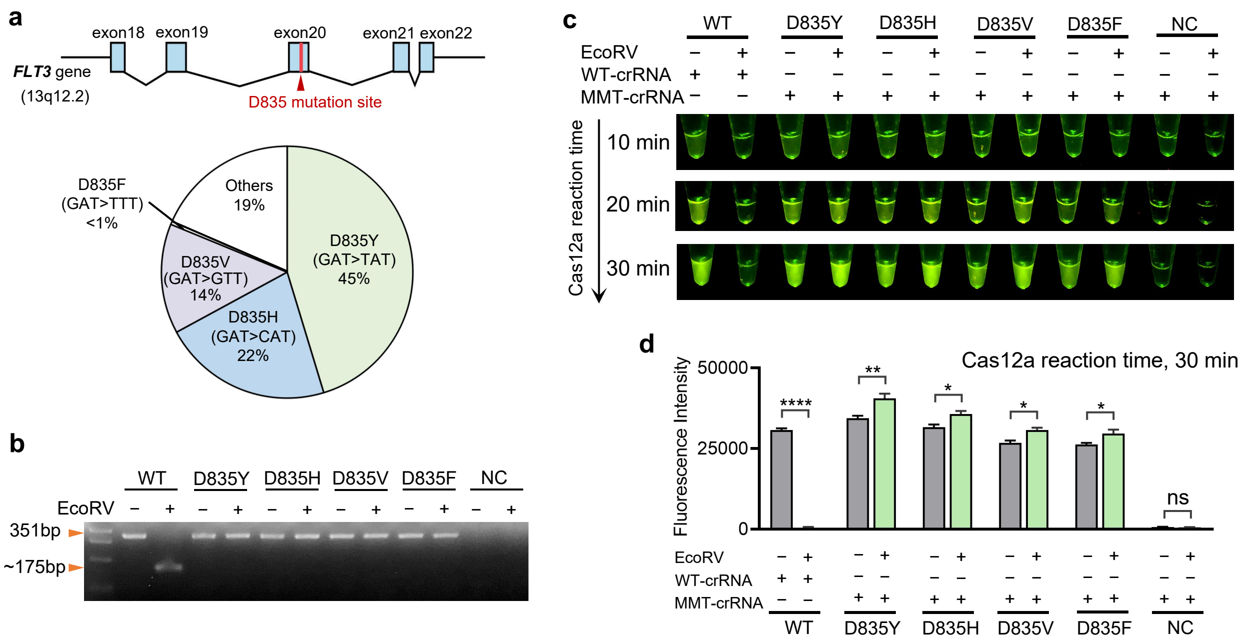

Fig. S1 Validation of the activity and specificity of EcoRV in RPA system. **a** The genomic location of *FLT3*-D835 mutation site and the proportions of different *FLT3*-D835 mutation types in AML patients in cBioportal database. **b** Activity and specificity test of EcoRV digestion in its specific buffer using 100% WT and 100% D835Y/H/V/F PCR fragments. **c** Activity and specificity test of EcoRV digestion in RPA mixture. 5e10 copies of 100% WT and 100% D835Y/H/V/F PCR fragments were treated in the RPA mixture without primers, at 37°C for 20 min, and then detected by Cas12a reaction. **d** Fluorescence intensity of (c) after Cas12a reaction for 30 min .

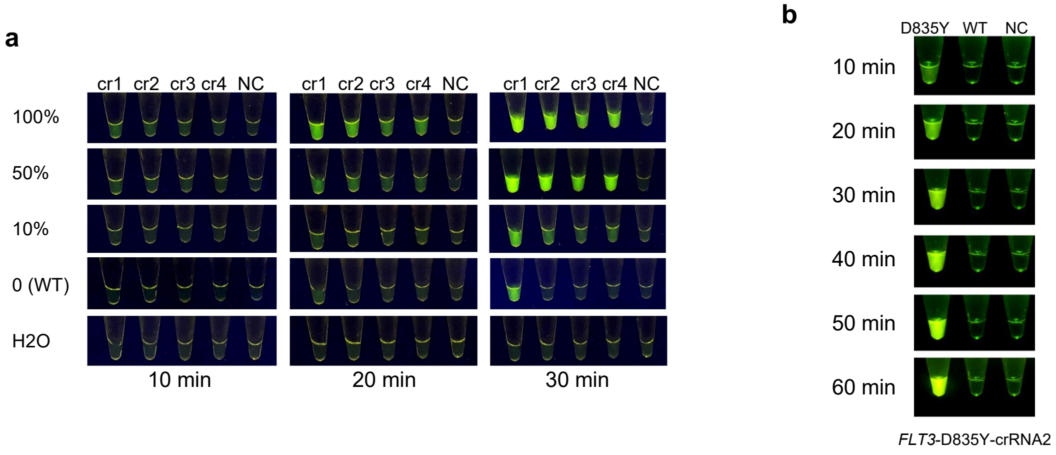

Fig. S2 Specificity assay of *FLT3*-D835Y-crRNAs. **a** Time-course analysis of *FLT3*-D835Y-crRNA1~4 guided Cas12a specific detection of the target PCR fragments with D835Y (GAT>TAT) mutation rate of 100%, 50%, 10%, 0 (WT), respectively. cr, crRNA; NC, negative control. **b** Time-course analysis of *FLT3*-D835Y-crRNA2 induced Cas12a specific reaction with the target *FLT3*-D835Y, WT, and NC fragments.

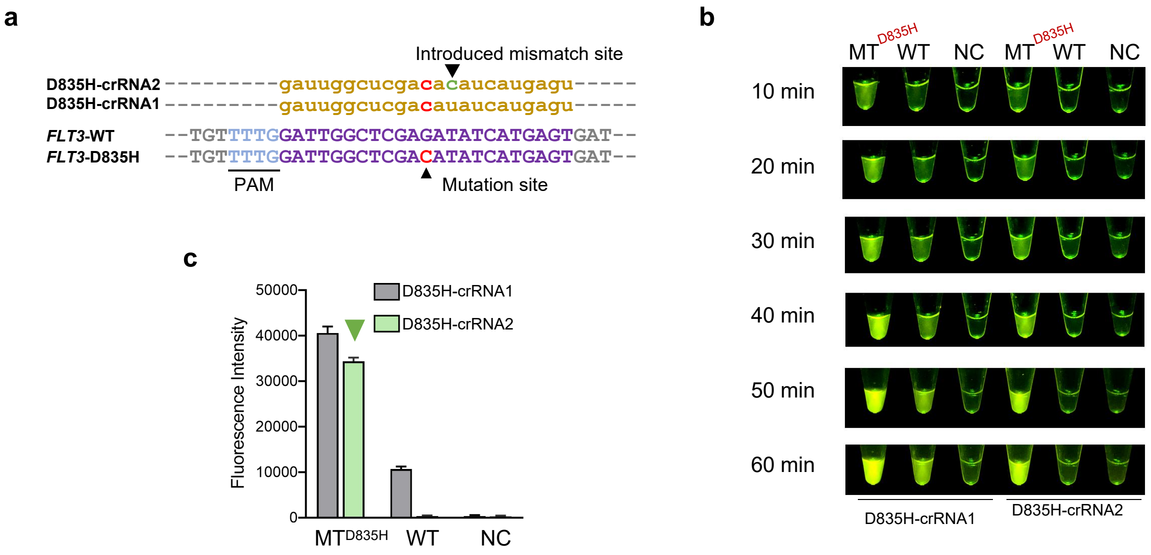

Fig. S3 Specificity assay of *FLT3*-D835H-crRNAs. **a** Sequences (brown) and target (purple) of *FLT3*-D835H-crRNA1 and crRNA2. The mutation base (G>C) and the introduced mismatch (U>C) are colored in red and green, respectively. **b** Time-course analysis of *FLT3*-D835H-crRNA1 and crRNA2 to detect a mutation between mutant (MT^{D835H}) and WT allele. **c**, Comparison of fluorescence intensity after 60 min reaction.

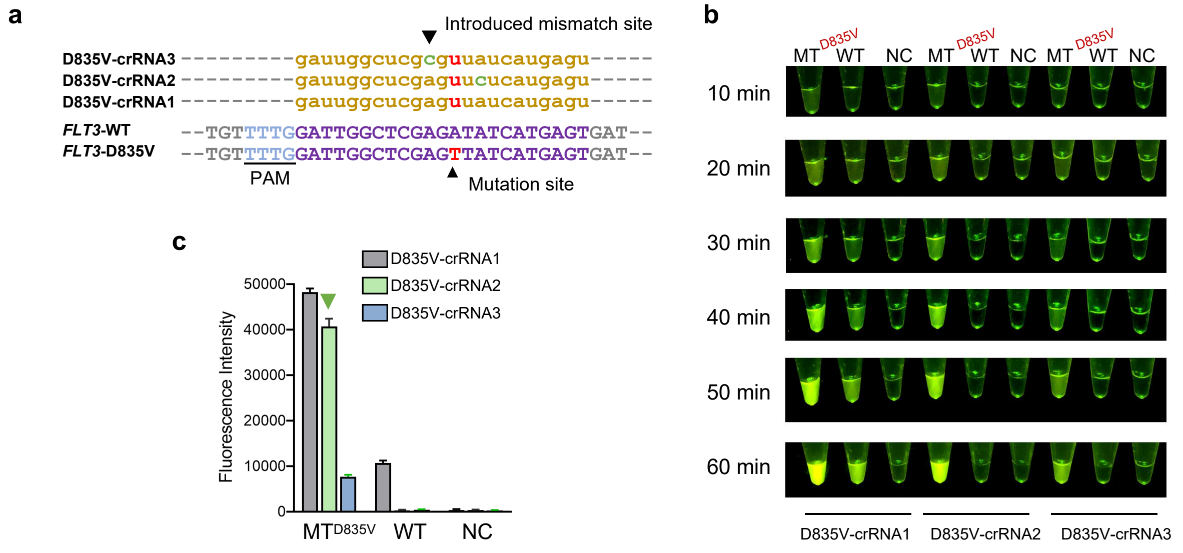

Fig. S4 Specificity assay of *FLT3*-D835V-crRNAs. **a** Sequences (brown) and target (purple) of *FLT3*-D835V-crRNA1~3. The mutation base (GAT>GTT) and the introduced mismatches are colored in red and green, respectively. **b** Time-course analysis of *FLT3*-D835V-crRNA1~3 to detect a mutation between mutant (MT^{D835V}) and WT allele. **c** Comparison of fluorescence intensity after 60 min reaction.

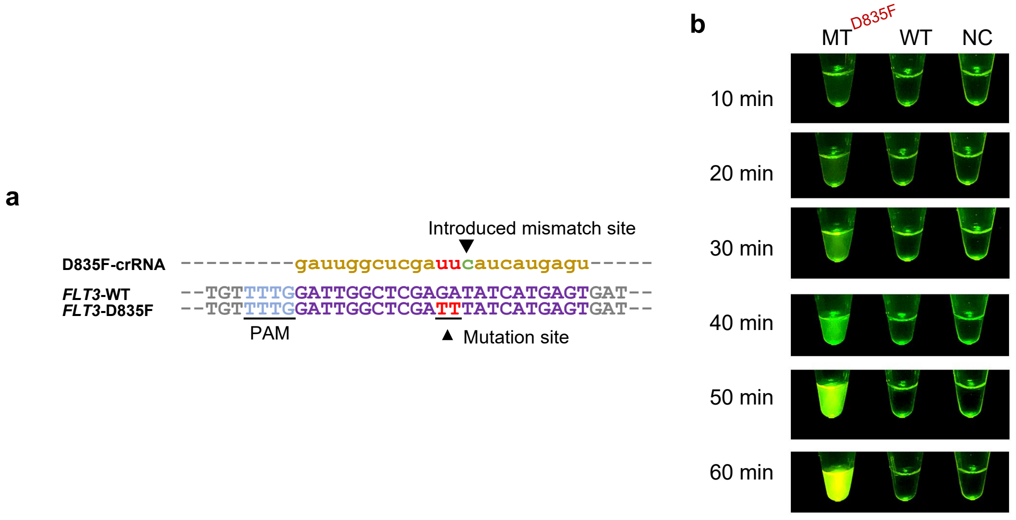

Fig. S5 Specificity assay of *FLT3*-D835F-crRNAs. **a** Sequences (brown) and target (purple) of *FLT3*-D835F-crRNA. The mutation bases (GAT>TTT) and an introduced mismatch are colored in red and orange, respectively. **b** Time-course analysis of *FLT3*-D835F-crRNA to detect the mutation between mutant (MT^{D835F}) and WT allele.

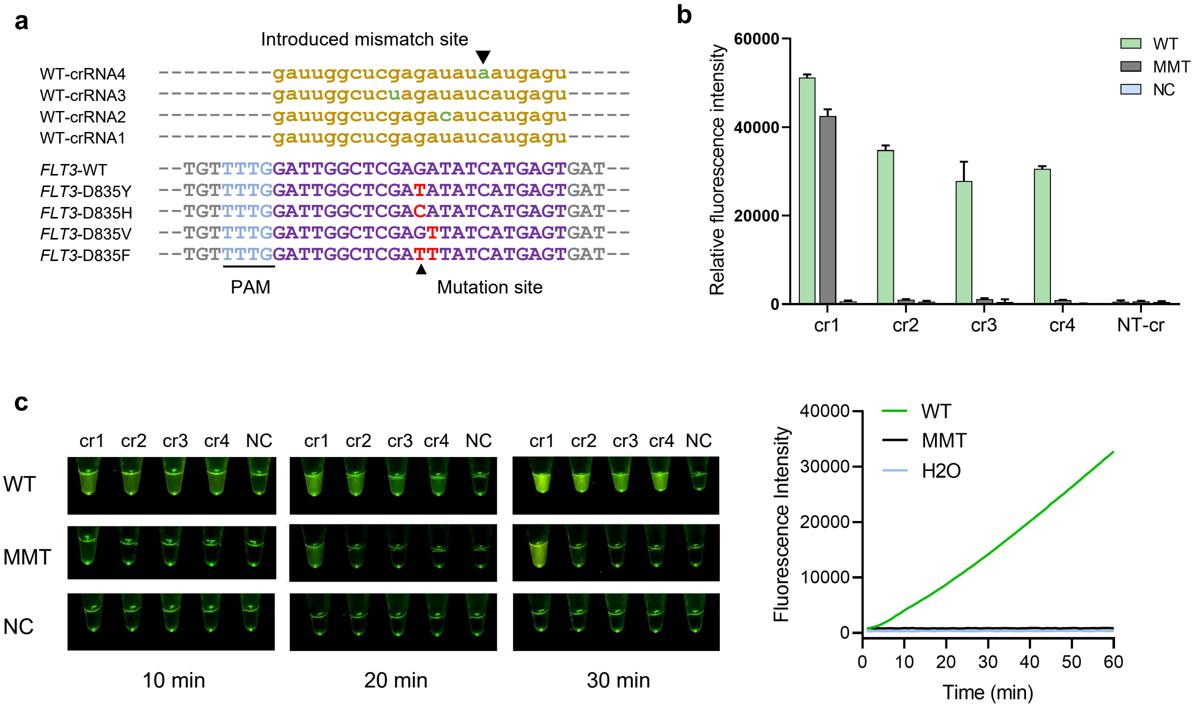

Fig. S6 Specificity assay of *FLT3*-D835WT-crRNAs. **a** Sequences (brown) and target (purple) of *FLT3*-D835-WT-crRNA1~4. The mutation bases of D835Y, D835H, D835V, and D835F are colored in red, and the introduced mismatches are colored in orange. **b** Comparison of fluorescence intensity after 60 min reaction, MMT = mixed-D835Y&H&V&F mutations. **c** Time-course analysis of *FLT3*-D835WT-crRNA1~4 to detect mutations between WT and mutant allele.

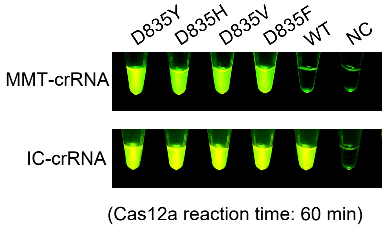

Fig. S7 Specificity assay of MMT-crRNAs. Specificity assay of MMT-crRNAs using 1e10 copies of D835Y/H/V/F and WT fragments. Photos were taken after 60 min of Cas12a reaction under a blue lamp.

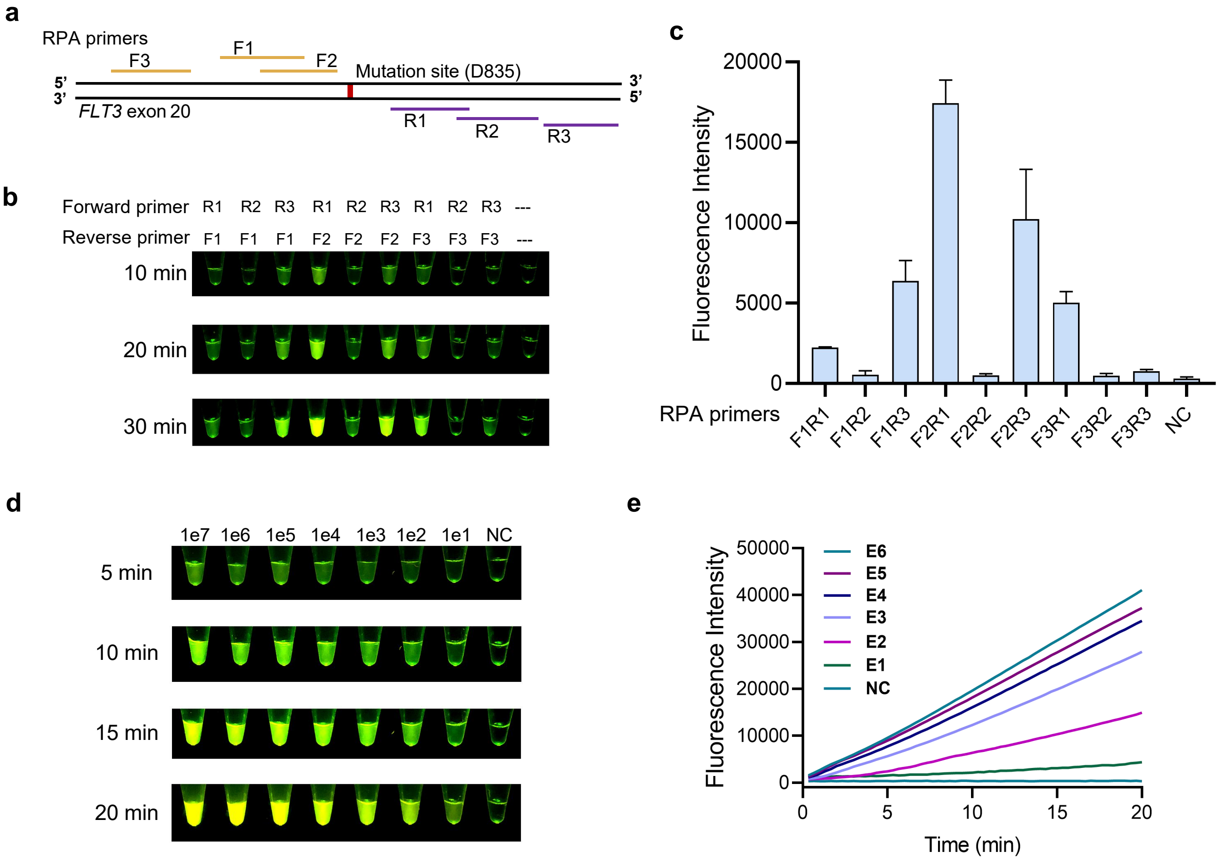

Fig. S8 RPA primer screen for highly sensitive EasyCatch detection. **a** Relative locations of designed RPA primers to the D835 site. **b** Time-course analysis of RPA primers screen for the amplification of *FLT3*-D835 region. The tested sample was 1e2 copies of 100% D835Y plasmid templates. And the reaction conditions were standard RPA for 20 min without EcoRV digestion, and MMT-crRNA-induced Cas12a reaction for 20 min, both under 37 °C. **c** Comparison of the final fluorescence intensity. **d** Amplification capacity test of F2R1-mediated RPA using gradient copies of D835Y templates and MMT-crRNAs-induced Cas12a reaction. **e** Dynamic analysis of (d).

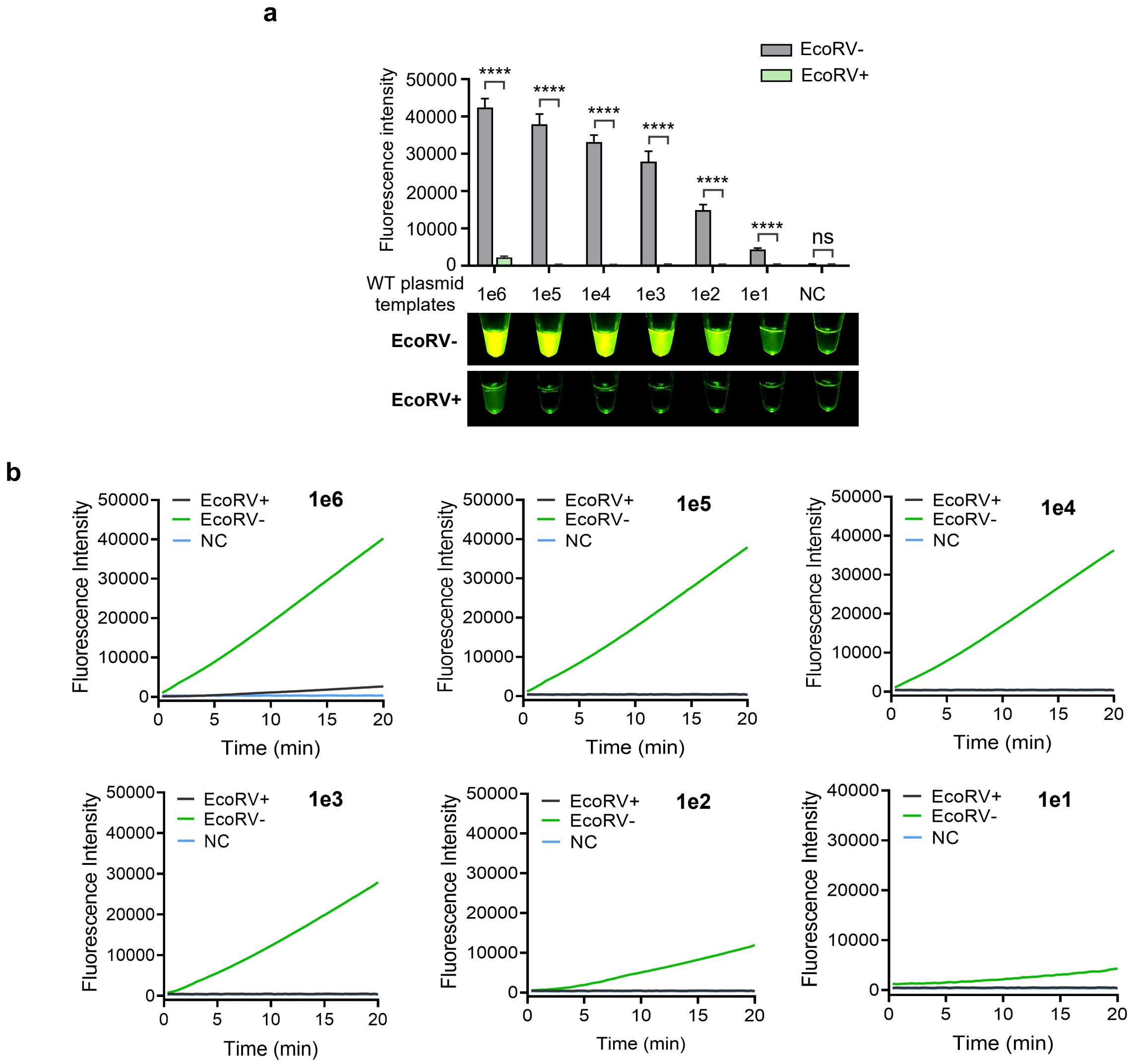

Fig. S9 WT inhibition assay by RPA with or without EcoRV and WT-crRNA induced Cas12a reaction, using 1e6 ~ 1e1 copies of WT plasmid templates. **a** Fluorescence intensity statistics and naked-eye results. **b** Time-course analysis.

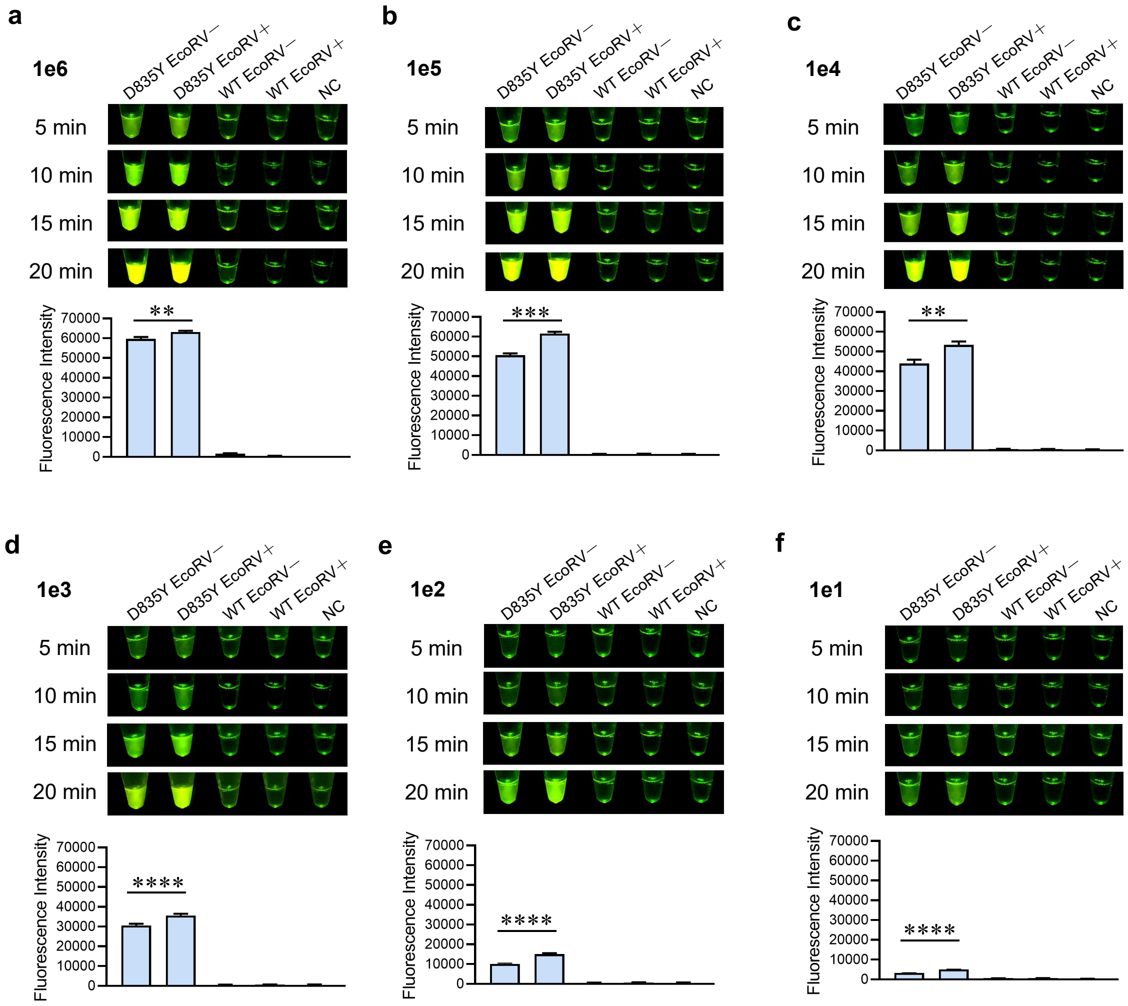

Fig. S10 Detection of 1e6 ~ 1e1 copies of D835Y and WT plasmids using RPA with or without EcoRV, combined with MMT-crRNA induced Cas12a reaction. Histograms show the final fluorescence intensity.

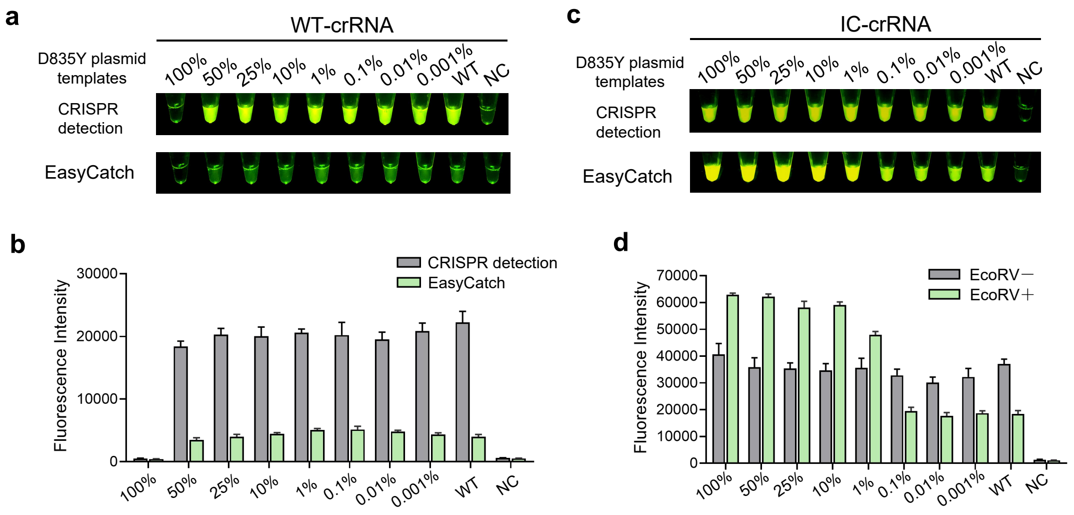

Fig. S11 Inhibition of WT amplification by EasyCatch. **a, b** Detection of 1e6 plasmid templates with gradient D835Y mutation rates using WT-crRNA-mediated CRISPR detection and EasyCatch. **c**, **d** Detection of 1e6 plasmid templates with gradient D835Y mutation rates using IC-crRNA-mediated CRISPR detection and EasyCatch.

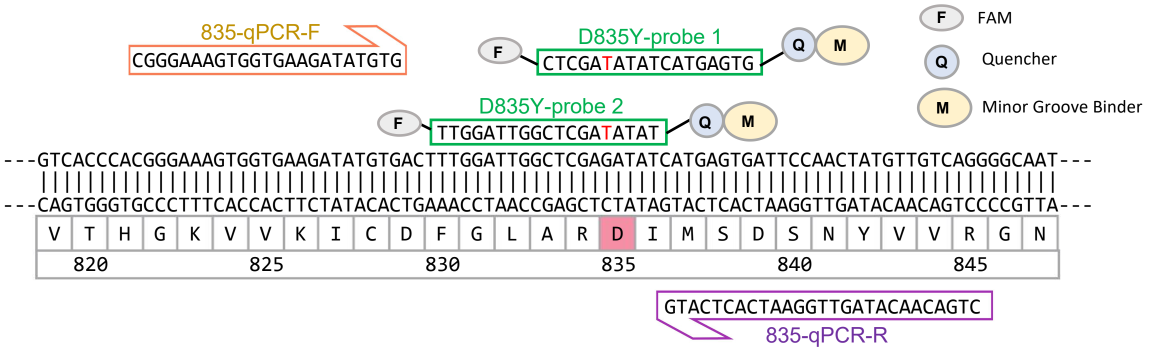

Fig. S12 Design of TaqMan qPCR for the detection of *FLT3*-D835Y. The forward primer, reverse primer and TaqMan probes are colored in orange, purple, and green, respectively.

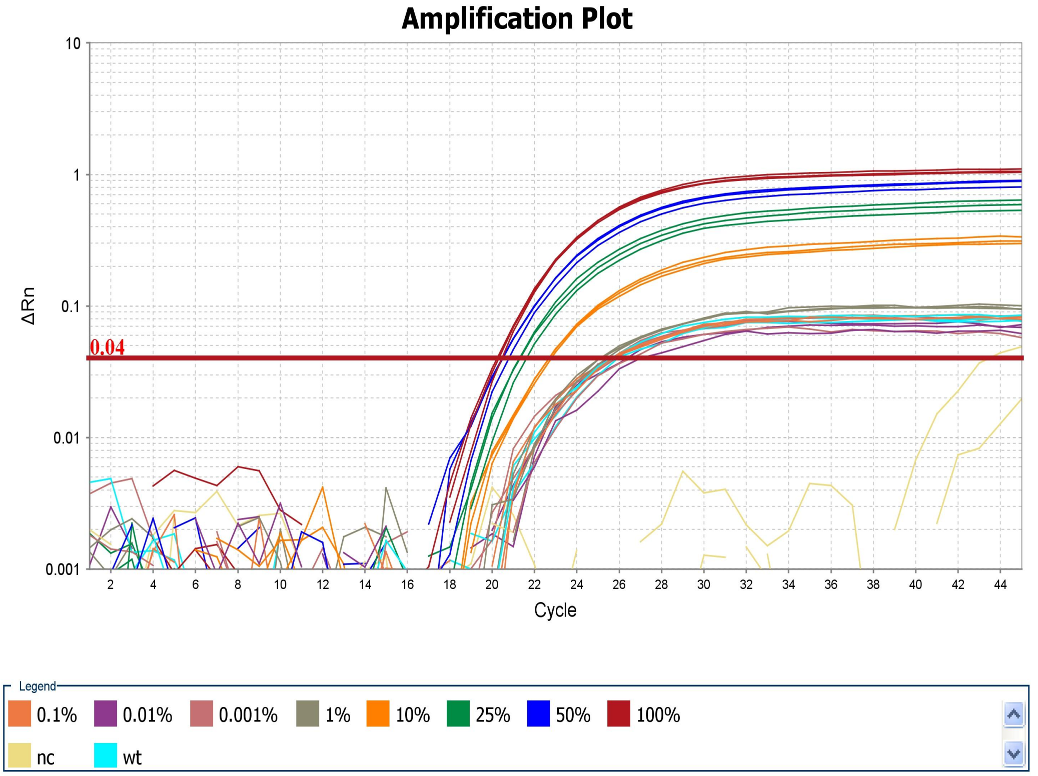

Fig. S13 The amplification plot of D835Y-probe 1-involved qPCR in the detection of 1e5 copies of plasmid templates with gradient D835Y mutation rates.

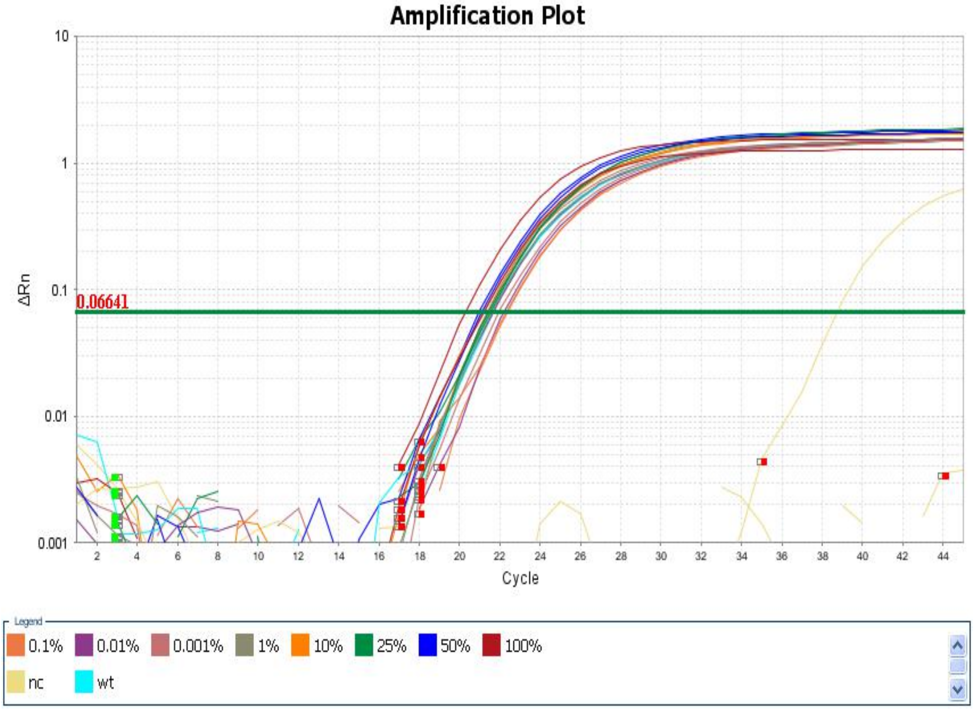

Fig. S14 The amplification plot of D835Y-probe 2-involved qPCR in the detection of 1e5 copies of plasmid templates with gradient D835Y mutation rates.
