# Supplementary material for: EasyCatch, a convenient, sensitive and specific CRISPR detection system for cancer gene mutations
Source: Mol Cancer. 2021 Dec 2;20:157. doi: 10.1186/s12943-021-01456-x (PMC8638196; doi:10.1186/s12943-021-01456-x)
Supplement: Supplementary file 3 — Additional file 3: Supplementary Fig. 15 Exploration of the fastest blood processing time before EasyCatch. Supplementary Fig. 16 Classification information and NGS results of the FLT3-D835 mutation status of 32 AML samples. Supplementary Fig. 17 EasyCatch and FGS results of the 32 AML samples in the detection of FLT3-D835Y/V/H/F nutations. Supplementary Fig. 18 Equipment needed in EasyCatch assay. Supplementary Fig. 19 EasyCatch results of 80 AML patient samples read by naked eyes. Supplementary Fig. 20 EasyCatch, FGS and NGS results of 80 AML patient blood samples. Supplementary Fig. 21 Broad application of EasyCatch in cancer mutation diagnosis. Supplementary Fig. 22 Comparison of CRISPR detection and EasyCatch on IDH2-R172K mutation. Supplementary Fig. 23 Comparison of CRISPR detection and EasyCatch on EGFR-e19del mutation. Supplementary Fig. 24 Comparison of CRISPR detection and EasyCatch on EGFR-L858R mutation. Supplementary Fig. 25 Comparison of CRISPR detection and EasyCatch on NRAS-G12D mutation. Supplementary Fig. 26 The amplification plot of fluorescence qPCR detection of EGFR gene e19del mutation using a commercial kit. Supplementary Fig. 27 The amplification plot of fluorescence qPCR detection of EGFR gene L858R mutation using a commercial kit. Supplementary Fig. 28 The amplification plot of fluorescence qPCR detection of EGFR gene L858R mutation using a commercial kit. Supplementary Fig. 29 The RPA primer design for IDH2-R172K mutation detection. [file 12943_2021_1456_MOESM3_ESM.pdf]

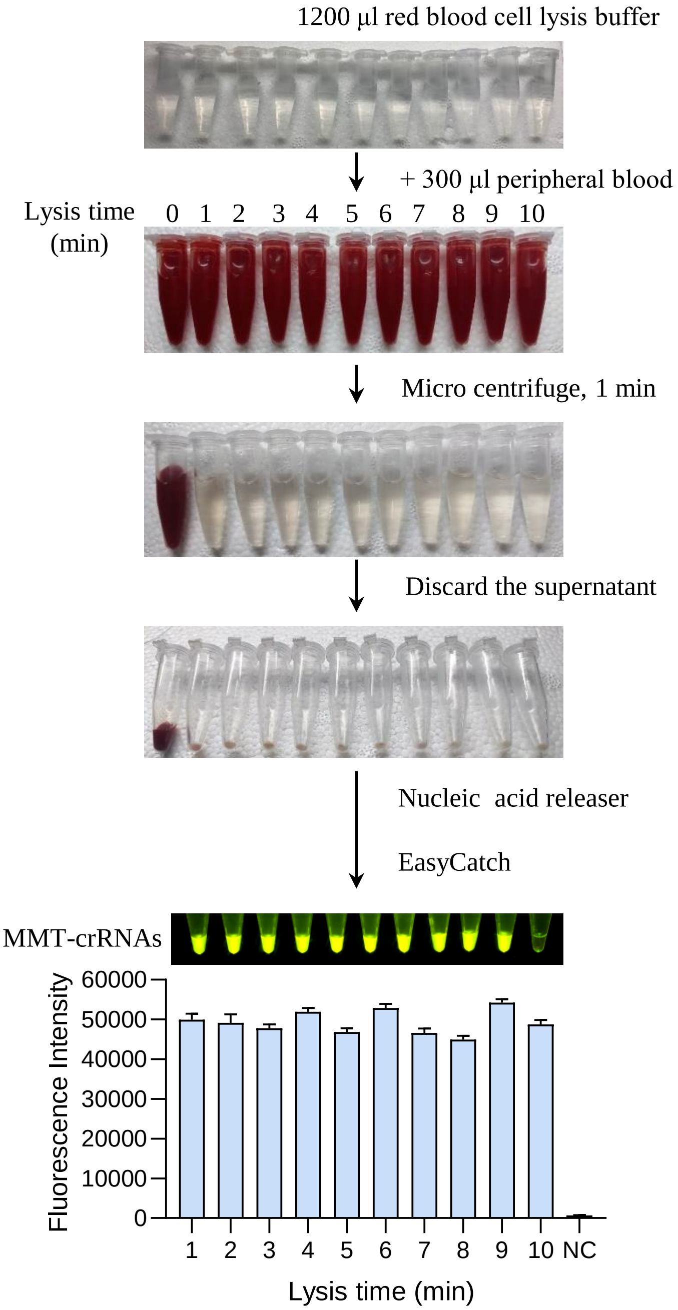

Fig. S15 Exploration of the fastest blood processing time before EasyCatch. Briefly, peripheral blood drawn from a patient with D835Y mutation was equally divided into 300 µl/sample, then mixed with 1200 µl red blood cell (RBC) lysis buffer. Different lysis time (0~10 min) was used to screen the fastest condition. Then white blood cells (WBC) were collected by centrifugal precipitation for 1 min, processed by nucleic acid releaser and detected using EasyCatch.

a

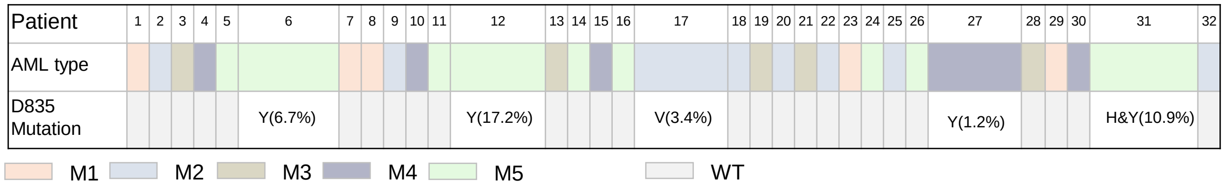

b

P1

G	100	0.0	0.0
A	0.0	100	0.0
C	0.0	0.0	0.0
T	0.1	0.0	100

P2

G	100	0.0	0.0
A	0.0	100	0.0
C	0.0	0.0	0.0
T	0.0	0.0	100

P3

G	100	0.0	0.0
A	0.0	100	0.0
C	0.0	0.0	0.0
T	0.2	0.0	100

P4

G	100	0.0	0.0
A	0.0	100	0.0
C	0.0	0.0	0.0
T	0.0	0.0	100

P5

G	100	0.0	0.0
A	0.0	100	0.0
C	0.0	0.0	0.0
T	0.1	0.0	100

P6

G	93.3	0.0	0.0
A	0.0	100	0.0
C	0.0	0.0	0.0
T	6.7	0.0	100

P7

G	100	0.0	0.0
A	0.0	100	0.0
C	0.0	0.0	0.0
T	0.0	0.0	100

P8

G	100	0.0	0.0
A	0.0	100	0.0
C	0.0	0.0	0.0
T	0.3	0.0	100

P9

G	100	0.0	0.0
A	0.0	100	0.0
C	0.0	0.0	0.0
T	0.0	0.0	100

P10

G	100	0.0	0.0
A	0.0	100	0.0
C	0.0	0.0	0.0
T	0.1	0.0	100

P11

G	100	0.0	0.0
A	0.0	100	0.0
C	0.0	0.0	0.0
T	0.0	0.0	100

P12

G	82.8	0.0	0.0
A	0.0	100	0.0
C	0.0	0.0	0.0
T	17.2	0.0	100

P13

G	100	0.0	0.0
A	0.0	100	0.0
C	0.0	0.0	0.0
T	0.2	0.0	100

P14

G	100	0.0	0.0
A	0.0	100	0.0
C	0.0	0.0	0.0
T	0.2	0.0	100

P15

G	100	0.0	0.0
A	0.0	100	0.0
C	0.0	0.0	0.0
T	0.0	0.0	100

P16

G	100	0.0	0.0
A	0.0	100	0.0
C	0.0	0.0	0.0
T	0.1	0.0	100

P17

G	100	0.0	0.0
A	0.0	96.6	0.0
C	0.0	0.0	0.0
T	0.0	3.4	100

P18

G	100	0.0	0.0
A	0.0	100	0.0
C	0.0	0.0	0.0
T	0.0	0.0	100

P19

G	100	0.0	0.0
A	0.0	100	0.0
C	0.0	0.0	0.0
T	0.1	0.0	100

P20

G	100	0.0	0.0
A	0.0	100	0.0
C	0.0	0.0	0.0
T	0.2	0.0	100

P21

G	100	0.0	0.0
A	0.0	100	0.0
C	0.0	0.0	0.0
T	0.2	0.0	100

P22

G	100	0.0	0.0
A	0.0	100	0.0
C	0.2	0.0	0.0
T	0.1	0.0	100

P23

G	100	0.0	0.0
A	0.0	100	0.0
C	0.0	0.0	0.0
T	0.0	0.0	100

P24

G	100	0.0	0.0
A	0.0	100	0.0
C	0.0	0.0	0.0
T	0.0	0.0	100

P25

G	100	0.0	0.0
A	0.0	100	0.0
C	0.2	0.0	0.0
T	0.0	0.1	100

P26

G	100	0.0	0.0
A	0.0	100	0.0
C	0.0	0.0	0.0
T	0.0	0.0	100

P27

G	98.8	0.0	0.0
A	0.0	100	0.0
C	0.0	0.0	0.0
T	1.2	0.0	100

P28

G	100	0.0	0.0
A	0.0	100	0.0
C	0.0	0.0	0.0
T	0.0	0.0	100

P29

G	100	0.0	0.0
A	0.0	100	0.0
C	0.0	0.0	0.0
T	0.1	0.0	100

P30

G	100	0.0	0.0
A	0.0	100	0.0
C	0.0	0.0	0.0
T	0.3	0.0	100

P31

G	89.1	0.0	0.0
A	0.0	100	0.0
C	8.0	0.0	0.0
T	2.8	0.0	100

P32

G	100	0.0	0.0
A	0.0	100	0.0
C	0.0	0.0	0.0
T	0.0	0.0	100

Fig. S16 Classification information (**a**) and NGS results of the *FLT3*-D835 mutation status (**b**) of 32 AML samples.

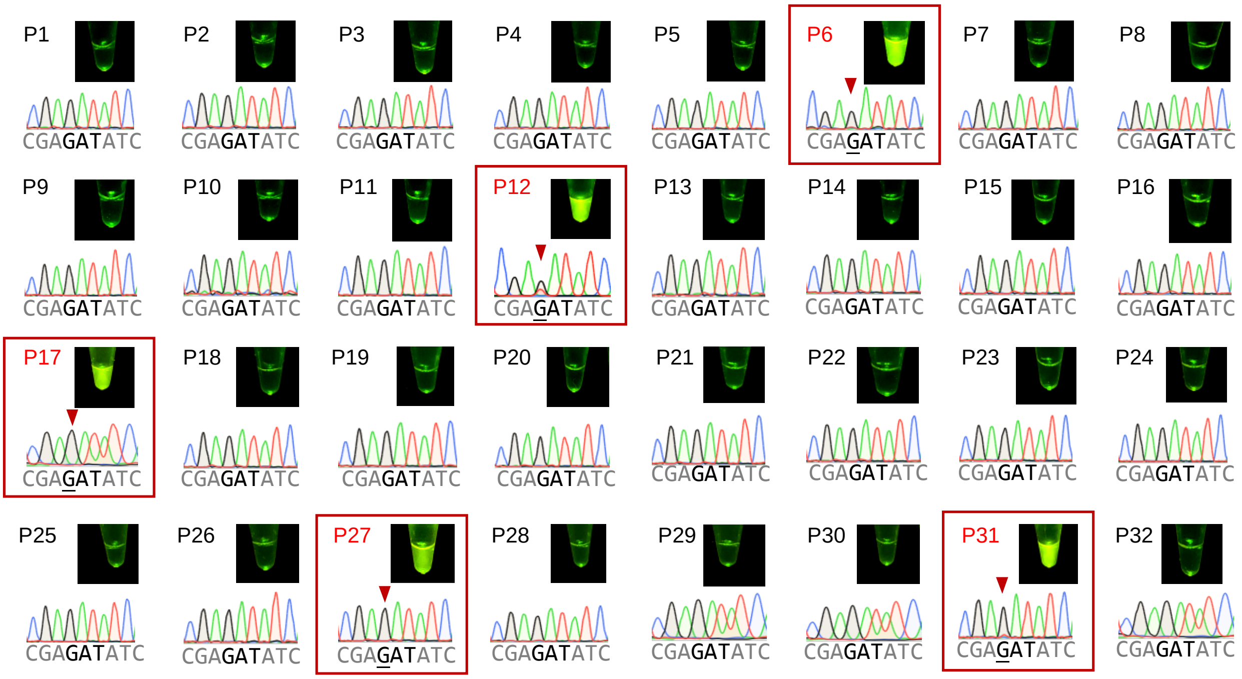

Fig. S17 EasyCatch and FGS results of the 32 AML samples in the detection of *FLT3*-D835Y/V/H/F nutations. Patients with mutations are marked by red IDs and red boxes, and red triangles indicate mutant bases.

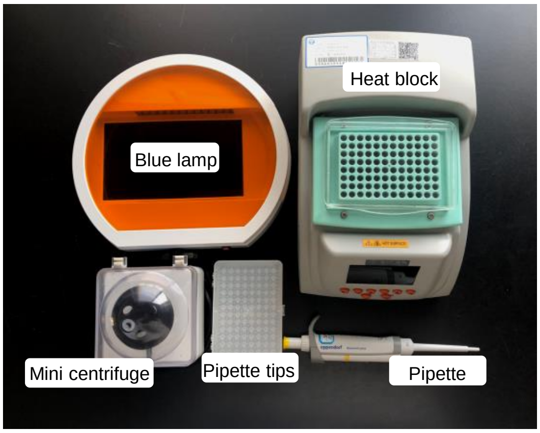

Fig. S18 Equipment needed in EasyCatch assay.

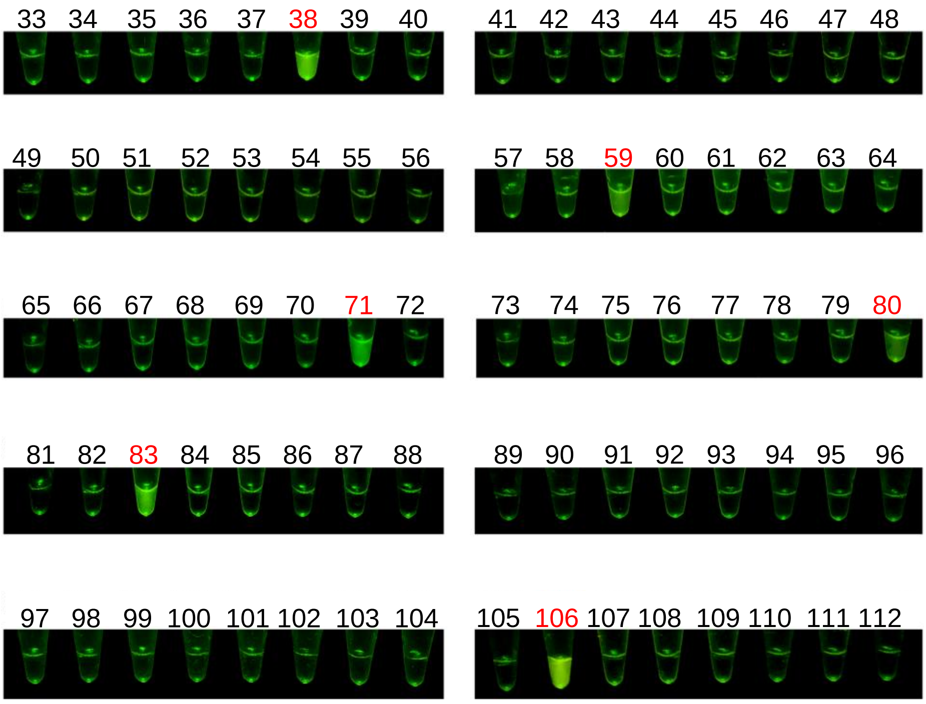

Fig. S19 EasyCatch results of 80 AML patient samples read by naked eyes under a 485 nm blue lamp.

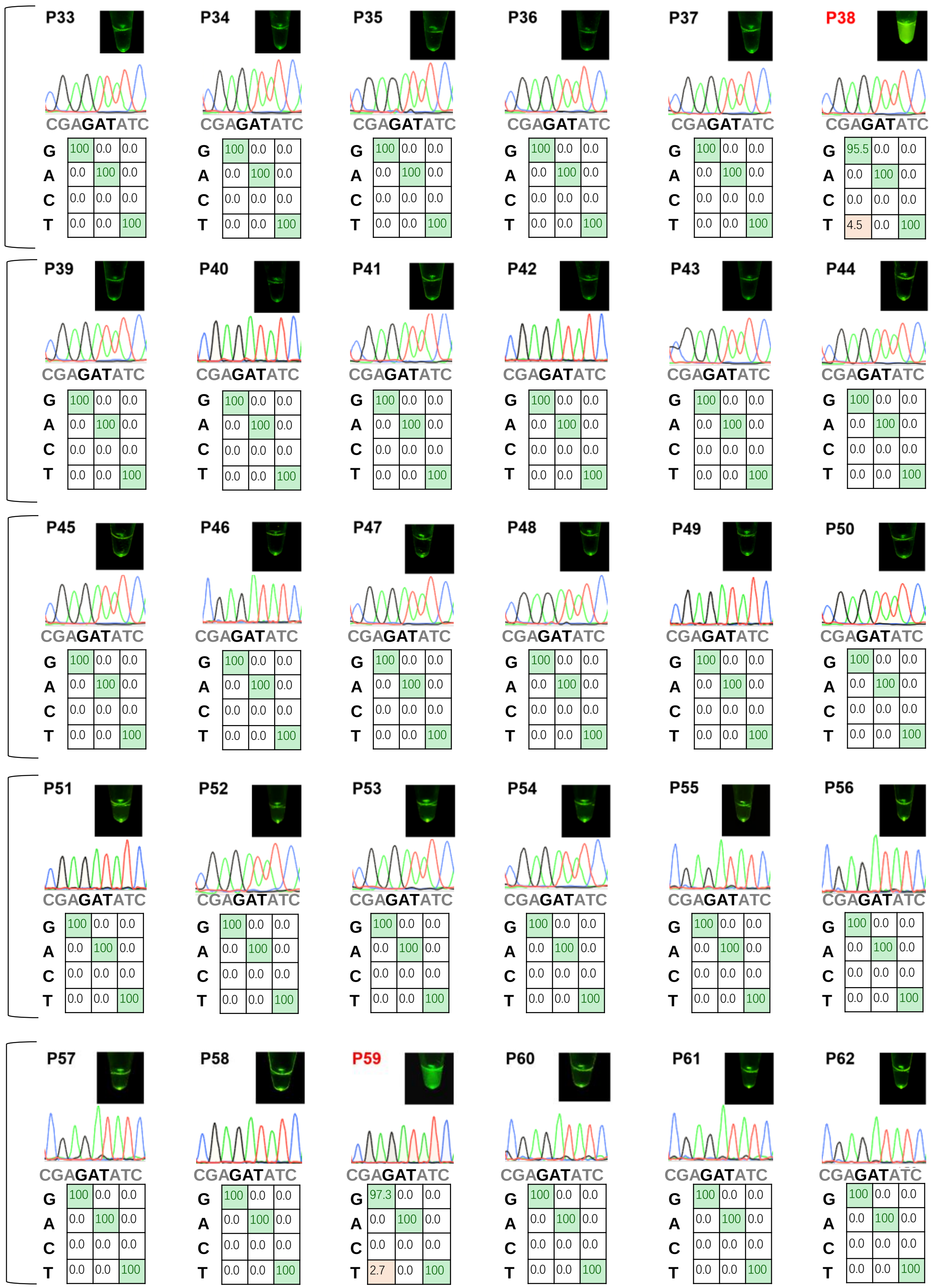
P33
CGAGATATC
G 100 0.0 0.0
A 0.0 100 0.0
C 0.0 0.0 0.0
T 0.0 0.0 100
P34
CGAGATATC
G 100 0.0 0.0
A 0.0 100 0.0
C 0.0 0.0 0.0
T 0.0 0.0 100
P35
CGAGATATC
G 100 0.0 0.0
A 0.0 100 0.0
C 0.0 0.0 0.0
T 0.0 0.0 100
P36
CGAGATATC
G 100 0.0 0.0
A 0.0 100 0.0
C 0.0 0.0 0.0
T 0.0 0.0 100
P37
CGAGATATC
G 100 0.0 0.0
A 0.0 100 0.0
C 0.0 0.0 0.0
T 0.0 0.0 100
P38
CGAGATATC
G 95.5 0.0 0.0
A 0.0 100 0.0
C 0.0 0.0 0.0
T 4.5 0.0 100
P39
CGAGATATC
G 100 0.0 0.0
A 0.0 100 0.0
C 0.0 0.0 0.0
T 0.0 0.0 100
P40
CGAGATATC
G 100 0.0 0.0
A 0.0 100 0.0
C 0.0 0.0 0.0
T 0.0 0.0 100
P41
CGAGATATC
G 100 0.0 0.0
A 0.0 100 0.0
C 0.0 0.0 0.0
T 0.0 0.0 100
P42
CGAGATATC
G 100 0.0 0.0
A 0.0 100 0.0
C 0.0 0.0 0.0
T 0.0 0.0 100
P43
CGAGATATC
G 100 0.0 0.0
A 0.0 100 0.0
C 0.0 0.0 0.0
T 0.0 0.0 100
P44
CGAGATATC
G 100 0.0 0.0
A 0.0 100 0.0
C 0.0 0.0 0.0
T 0.0 0.0 100
P45
CGAGATATC
G 100 0.0 0.0
A 0.0 100 0.0
C 0.0 0.0 0.0
T 0.0 0.0 100
P46
CGAGATATC
G 100 0.0 0.0
A 0.0 100 0.0
C 0.0 0.0 0.0
T 0.0 0.0 100
P47
CGAGATATC
G 100 0.0 0.0
A 0.0 100 0.0
C 0.0 0.0 0.0
T 0.0 0.0 100
P48
CGAGATATC
G 100 0.0 0.0
A 0.0 100 0.0
C 0.0 0.0 0.0
T 0.0 0.0 100
P49
CGAGATATC
G 100 0.0 0.0
A 0.0 100 0.0
C 0.0 0.0 0.0
T 0.0 0.0 100
P50
CGAGATATC
G 100 0.0 0.0
A 0.0 100 0.0
C 0.0 0.0 0.0
T 0.0 0.0 100
P51
CGAGATATC
G 100 0.0 0.0
A 0.0 100 0.0
C 0.0 0.0 0.0
T 0.0 0.0 100
P52
CGAGATATC
G 100 0.0 0.0
A 0.0 100 0.0
C 0.0 0.0 0.0
T 0.0 0.0 100
P53
CGAGATATC
G 100 0.0 0.0
A 0.0 100 0.0
C 0.0 0.0 0.0
T 0.0 0.0 100
P54
CGAGATATC
G 100 0.0 0.0
A 0.0 100 0.0
C 0.0 0.0 0.0
T 0.0 0.0 100
P55
CGAGATATC
G 100 0.0 0.0
A 0.0 100 0.0
C 0.0 0.0 0.0
T 0.0 0.0 100
P56
CGAGATATC
G 100 0.0 0.0
A 0.0 100 0.0
C 0.0 0.0 0.0
T 0.0 0.0 100
P57
CGAGATATC
G 100 0.0 0.0
A 0.0 100 0.0
C 0.0 0.0 0.0
T 0.0 0.0 100
P58
CGAGATATC
G 100 0.0 0.0
A 0.0 100 0.0
C 0.0 0.0 0.0
T 0.0 0.0 100
P59
CGAGATATC
G 97.3 0.0 0.0
A 0.0 100 0.0
C 0.0 0.0 0.0
T 2.7 0.0 100
P60
CGAGATATC
G 100 0.0 0.0
A 0.0 100 0.0
C 0.0 0.0 0.0
T 0.0 0.0 100
P61
CGAGATATC
G 100 0.0 0.0
A 0.0 100 0.0
C 0.0 0.0 0.0
T 0.0 0.0 100
P62
CGAGATATC
G 100 0.0 0.0
A 0.0 100 0.0
C 0.0 0.0 0.0
T 0.0 0.0 100

(Continued)

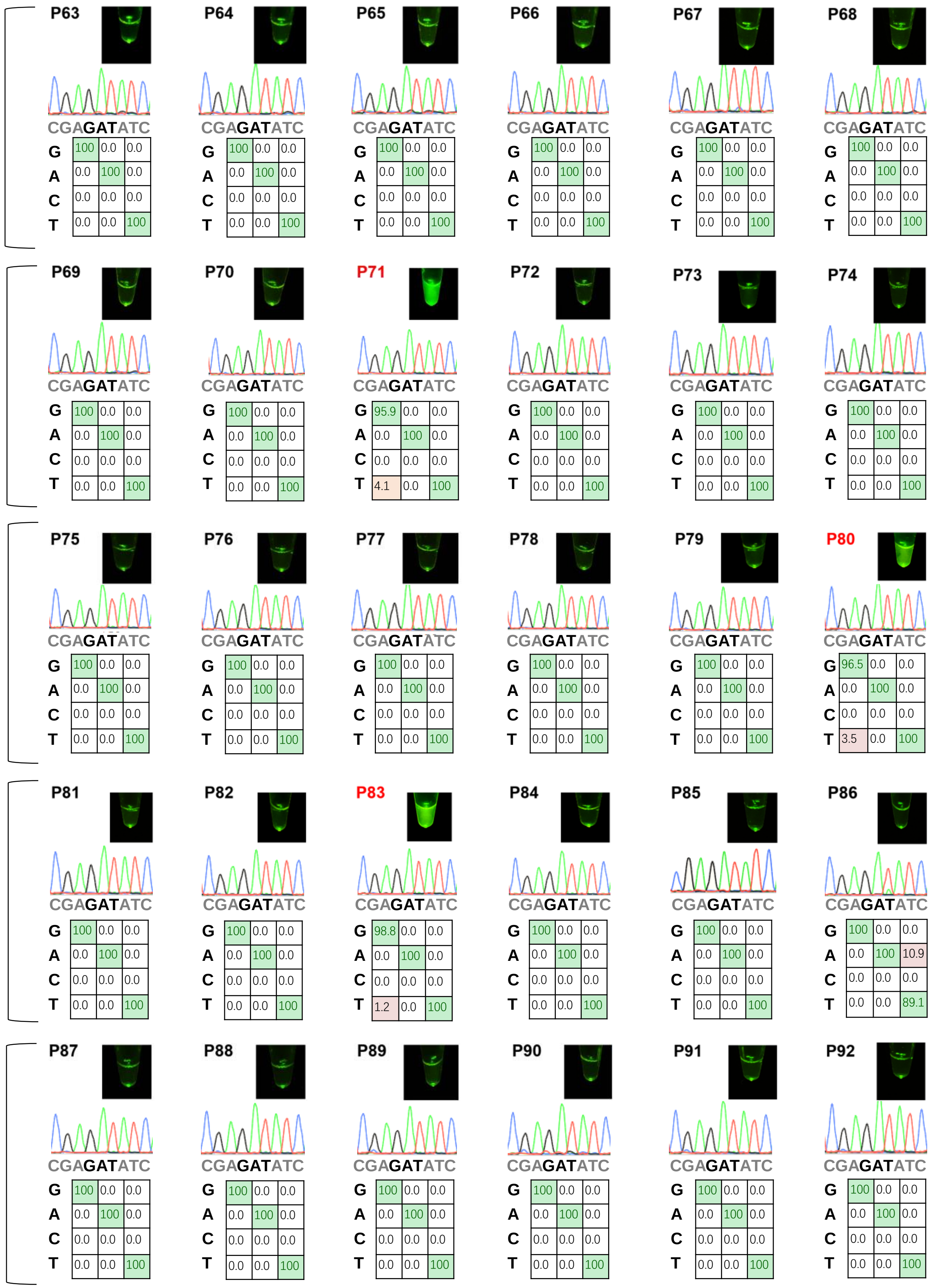
P63
CGAGATATC
G 100 0.0 0.0
A 0.0 100 0.0
C 0.0 0.0 0.0
T 0.0 0.0 100
P64
CGAGATATC
G 100 0.0 0.0
A 0.0 100 0.0
C 0.0 0.0 0.0
T 0.0 0.0 100
P65
CGAGATATC
G 100 0.0 0.0
A 0.0 100 0.0
C 0.0 0.0 0.0
T 0.0 0.0 100
P66
CGAGATATC
G 100 0.0 0.0
A 0.0 100 0.0
C 0.0 0.0 0.0
T 0.0 0.0 100
P67
CGAGATATC
G 100 0.0 0.0
A 0.0 100 0.0
C 0.0 0.0 0.0
T 0.0 0.0 100
P68
CGAGATATC
G 100 0.0 0.0
A 0.0 100 0.0
C 0.0 0.0 0.0
T 0.0 0.0 100
P69
CGAGATATC
G 100 0.0 0.0
A 0.0 100 0.0
C 0.0 0.0 0.0
T 0.0 0.0 100
P70
CGAGATATC
G 100 0.0 0.0
A 0.0 100 0.0
C 0.0 0.0 0.0
T 0.0 0.0 100
P71
CGAGATATC
G 95.9 0.0 0.0
A 0.0 100 0.0
C 0.0 0.0 0.0
T 4.1 0.0 100
P72
CGAGATATC
G 100 0.0 0.0
A 0.0 100 0.0
C 0.0 0.0 0.0
T 0.0 0.0 100
P73
CGAGATATC
G 100 0.0 0.0
A 0.0 100 0.0
C 0.0 0.0 0.0
T 0.0 0.0 100
P74
CGAGATATC
G 100 0.0 0.0
A 0.0 100 0.0
C 0.0 0.0 0.0
T 0.0 0.0 100
P75
CGAGATATC
G 100 0.0 0.0
A 0.0 100 0.0
C 0.0 0.0 0.0
T 0.0 0.0 100
P76
CGAGATATC
G 100 0.0 0.0
A 0.0 100 0.0
C 0.0 0.0 0.0
T 0.0 0.0 100
P77
CGAGATATC
G 100 0.0 0.0
A 0.0 100 0.0
C 0.0 0.0 0.0
T 0.0 0.0 100
P78
CGAGATATC
G 100 0.0 0.0
A 0.0 100 0.0
C 0.0 0.0 0.0
T 0.0 0.0 100
P79
CGAGATATC
G 100 0.0 0.0
A 0.0 100 0.0
C 0.0 0.0 0.0
T 0.0 0.0 100
P80
CGAGATATC
G 96.5 0.0 0.0
A 0.0 100 0.0
C 0.0 0.0 0.0
T 3.5 0.0 100
P81
CGAGATATC
G 100 0.0 0.0
A 0.0 100 0.0
C 0.0 0.0 0.0
T 0.0 0.0 100
P82
CGAGATATC
G 100 0.0 0.0
A 0.0 100 0.0
C 0.0 0.0 0.0
T 0.0 0.0 100
P83
CGAGATATC
G 98.8 0.0 0.0
A 0.0 100 0.0
C 0.0 0.0 0.0
T 1.2 0.0 100
P84
CGAGATATC
G 100 0.0 0.0
A 0.0 100 0.0
C 0.0 0.0 0.0
T 0.0 0.0 100
P85
CGAGATATC
G 100 0.0 0.0
A 0.0 100 0.0
C 0.0 0.0 0.0
T 0.0 0.0 100
P86
CGAGATATC
G 100 0.0 0.0
A 0.0 100 10.9
C 0.0 0.0 0.0
T 0.0 0.0 89.1
P87
CGAGATATC
G 100 0.0 0.0
A 0.0 100 0.0
C 0.0 0.0 0.0
T 0.0 0.0 100
P88
CGAGATATC
G 100 0.0 0.0
A 0.0 100 0.0
C 0.0 0.0 0.0
T 0.0 0.0 100
P89
CGAGATATC
G 100 0.0 0.0
A 0.0 100 0.0
C 0.0 0.0 0.0
T 0.0 0.0 100
P90
CGAGATATC
G 100 0.0 0.0
A 0.0 100 0.0
C 0.0 0.0 0.0
T 0.0 0.0 100
P91
CGAGATATC
G 100 0.0 0.0
A 0.0 100 0.0
C 0.0 0.0 0.0
T 0.0 0.0 100
P92
CGAGATATC
G 100 0.0 0.0
A 0.0 100 0.0
C 0.0 0.0 0.0
T 0.0 0.0 100

(Continued)

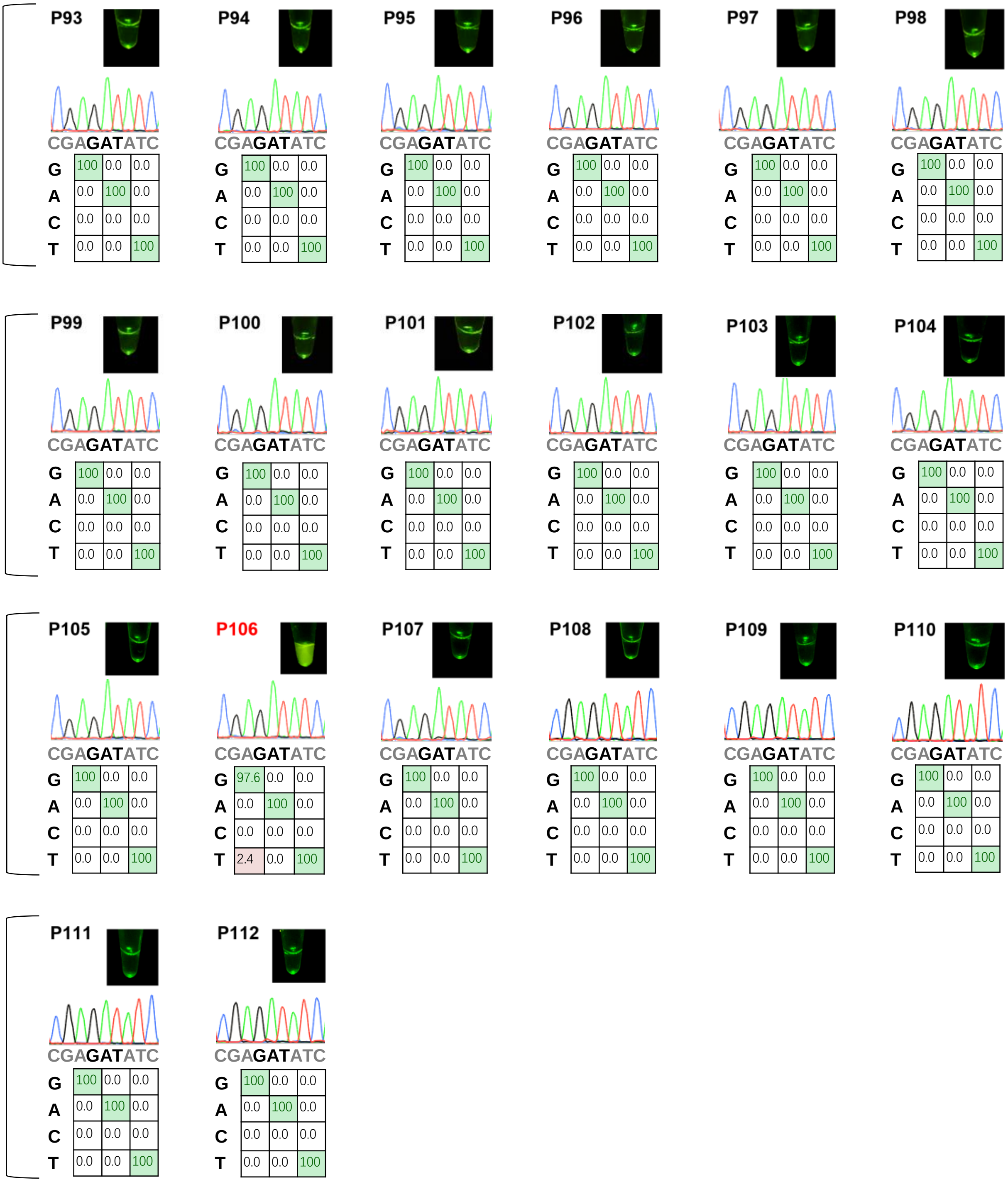

Fig. S20 EasyCatch, first-generation sequencing (FGS) and next-generation sequencing (NGS) results of 80 AML patient blood samples. Only three bases of D835 site are showed in NGS results, wild-type bases and mutated bases are colored in green and red, respectively.

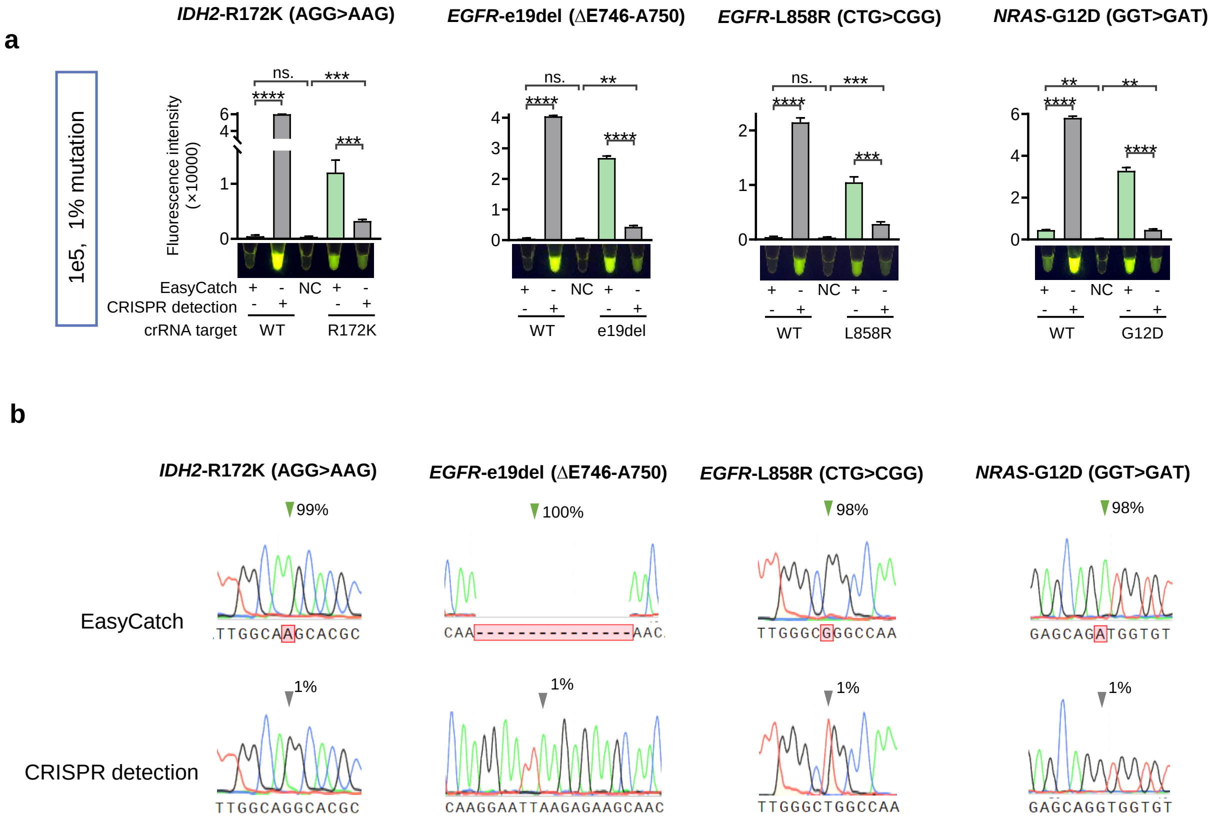

Fig. S21 Broad application of EasyCatch in cancer mutation diagnosis. **a** Sensitivity comparison between EasyCatch and CRISPR detection in the detection of *IDH2*-R172K, *EGFR*-e19del and L858R, and *NRAS*-G12D mutations. The tested samples were 1e5 copies of plasmid templates with a mutation rate of 1%. Each amplified product was detected by both WT-crRNA and mutation-crRNA induced Cas12a reaction. Fluorescence intensity and naked eye results were both recorded. **b** FGS results of the amplified products of EasyCatch and CRISPR detection. The tested samples were 1e5 copies of plasmid templates with a mutation rate of 1%.

IDH2-R172K (AGG>AAG)

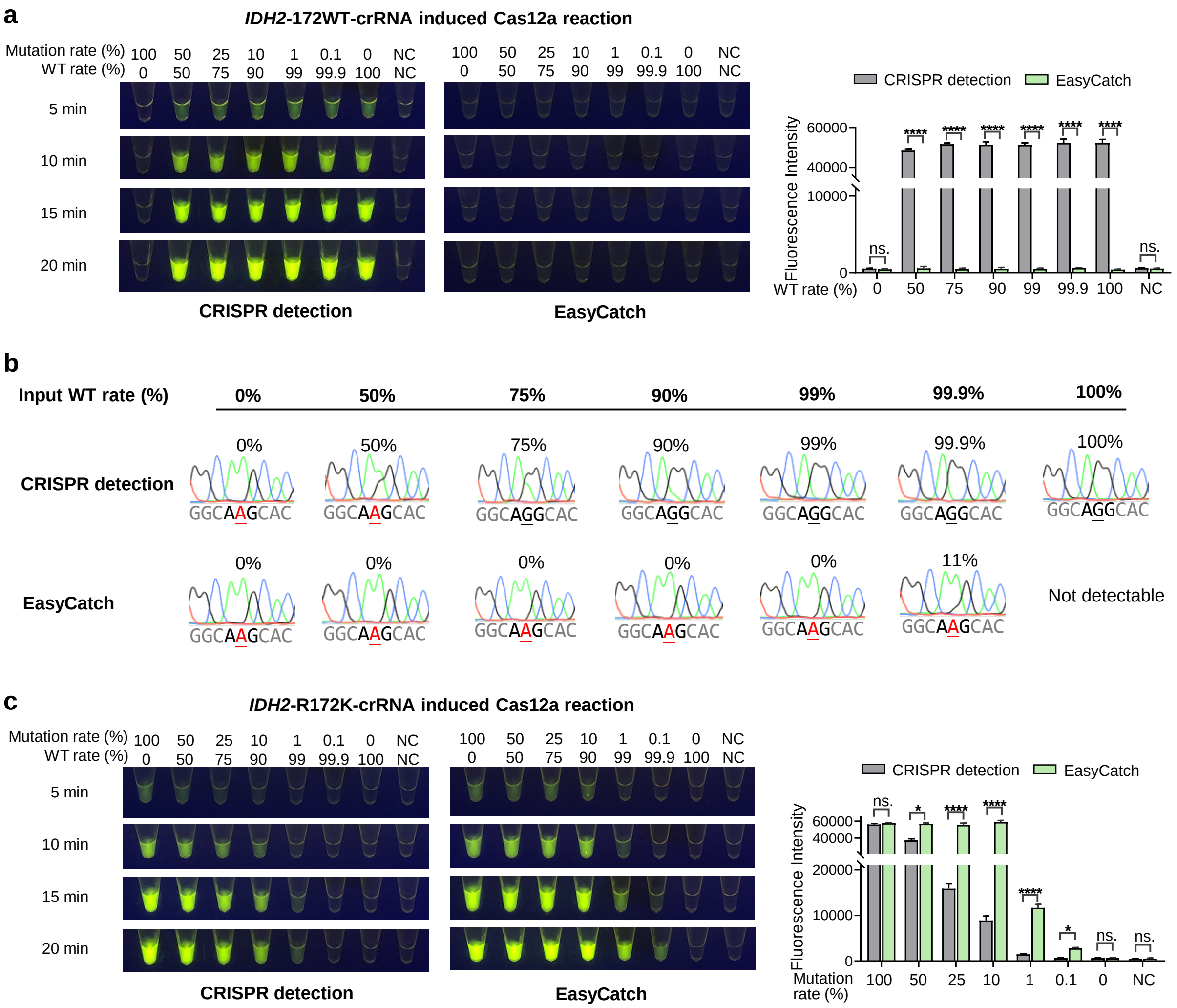

Fig. S22 Comparison of CRISPR detection and EasyCatch on *IDH2*-R172K mutation. **a** The 172WT-crRNA induced Cas12a reaction results of a series of products with different input mutation rates. A statistical analysis of reaction 20-min fluorescence intensity is on the right. **b** Comparison of output WT concentrations of products in CRISPR detection and EasyCatch by FGS. **c** The R172K-crRNA induced Cas12a reaction results of CRISPR detection and EasyCatch. A statistical analysis of reaction 20-min fluorescence intensity is on the right.

EGFR-e19del (15bp deletion)

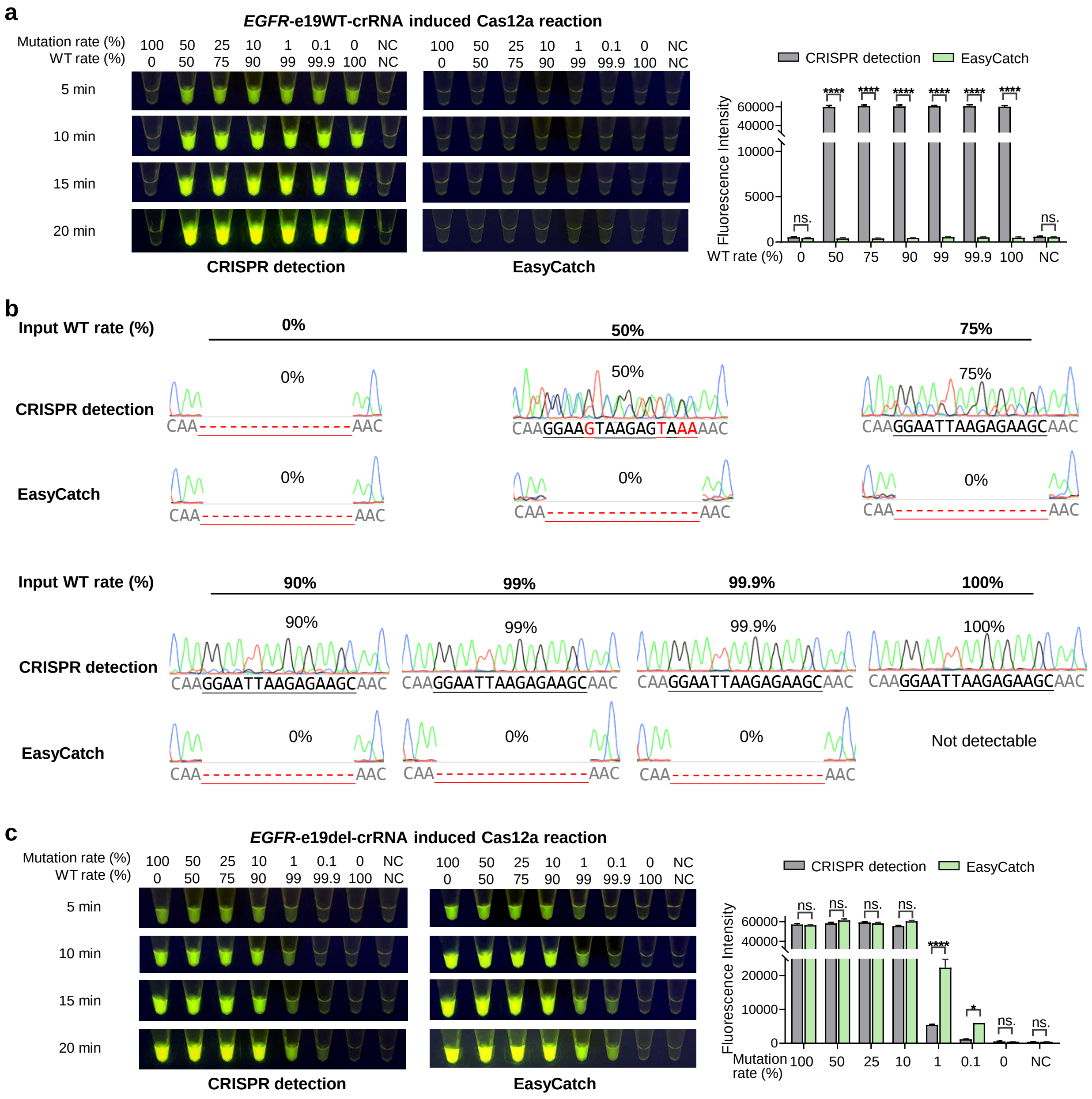

Fig. S23 Comparison of CRISPR detection and EasyCatch on *EGFR*-e19del mutation. **a** The e19WT-crRNA induced Cas12a reaction results of a series of products with different input mutation rates. A statistical analysis of reaction 20-min fluorescence intensity is on the right. **b** Comparison of output WT concentrations of products in CRISPR detection and EasyCatch by FGS. **c** The e19del-crRNA induced Cas12a reaction results of CRISPR detection and EasyCatch. A statistical analysis of reaction 20-min fluorescence intensity is on the right.

EGFR-L858R (CTG>CGG)

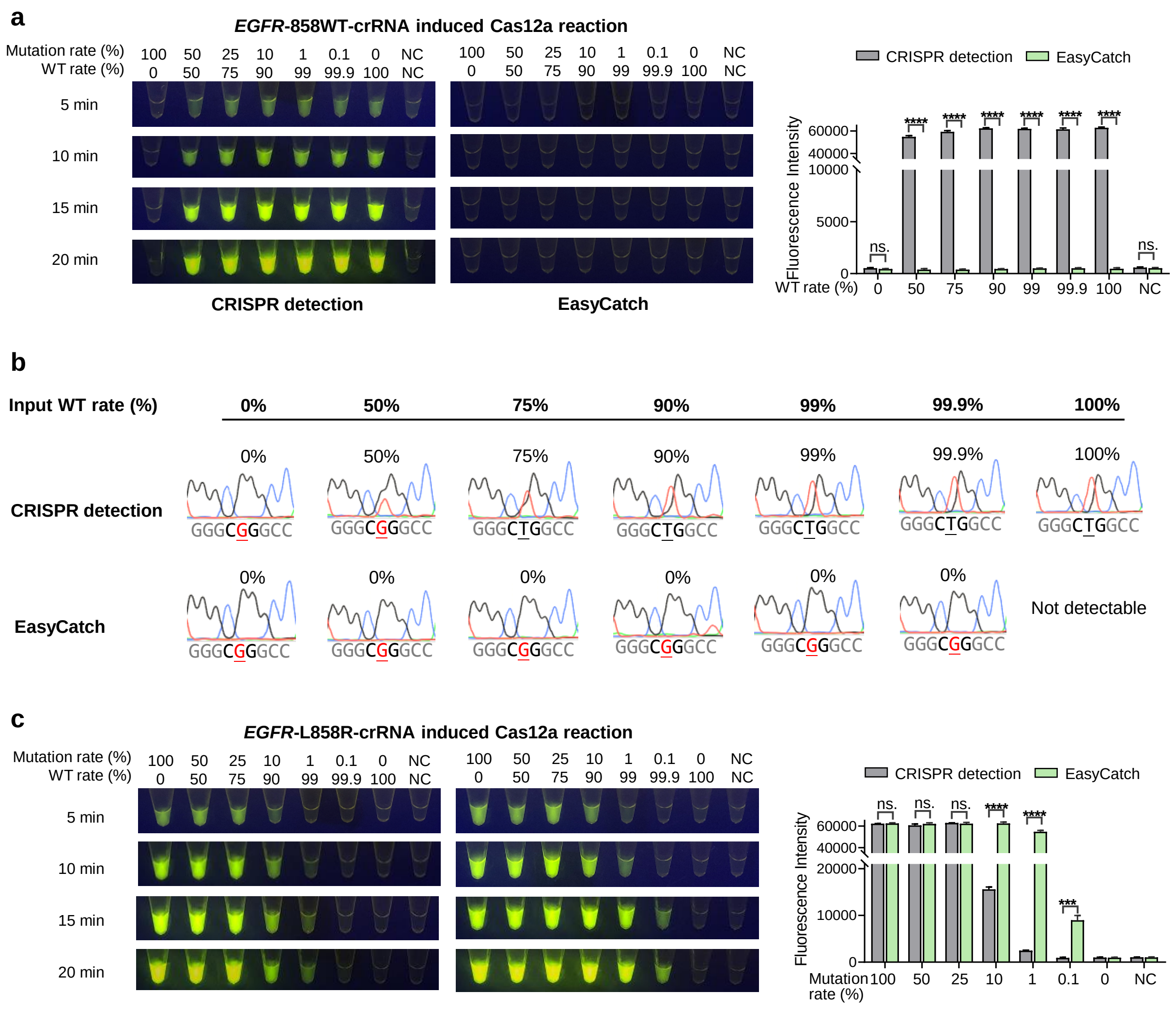

Fig. S24 Comparison of CRISPR detection and EasyCatch on *EGFR*-L858R mutation. **a** The 858WT-crRNA induced Cas12a reaction results of a series of products with different input mutation rates. A statistical analysis of reaction 20-min fluorescence intensity is on the right. **b** Comparison of output WT concentrations of products in CRISPR detection and EasyCatch by FGS. **c** The L858R-crRNA induced Cas12a reaction results of CRISPR detection and EasyCatch. A statistical analysis of reaction 20-min fluorescence intensity is on the right.

NRAS-G12D (GGT>GAT)

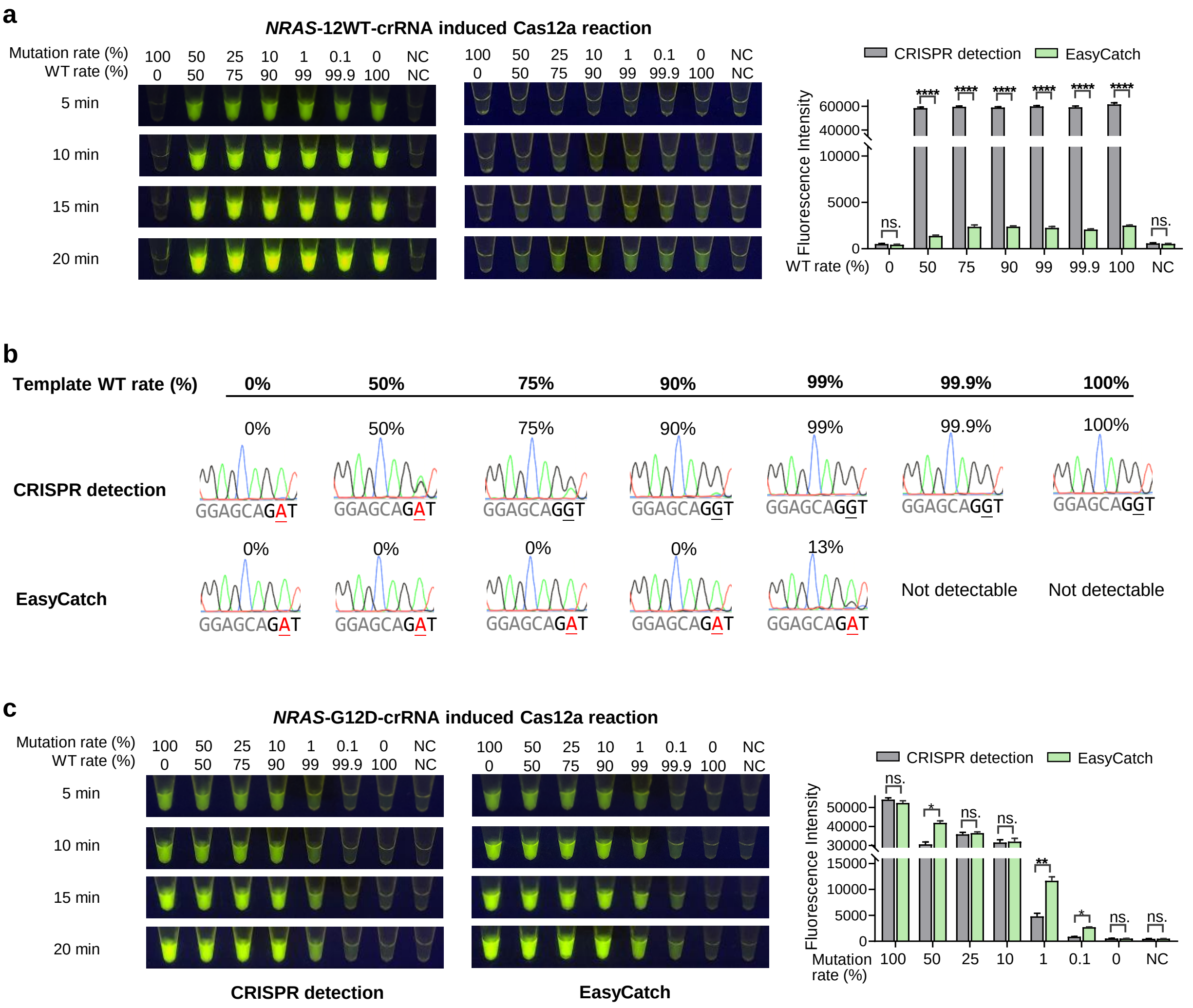

Fig. S25 Comparison of CRISPR detection and EasyCatch on *NRAS*-G12D mutation. **a** The 12WT-crRNA induced Cas12a reaction results of a series of products with different input mutation rates. A statistical analysis of reaction 20-min fluorescence intensity is on the right. **b** Comparison of output WT concentrations of products in CRISPR detection and EasyCatch by FGS. **c** The G12D-crRNA induced Cas12a reaction results of CRISPR detection and EasyCatch. A statistical analysis of reaction 20-min fluorescence intensity is on the right.

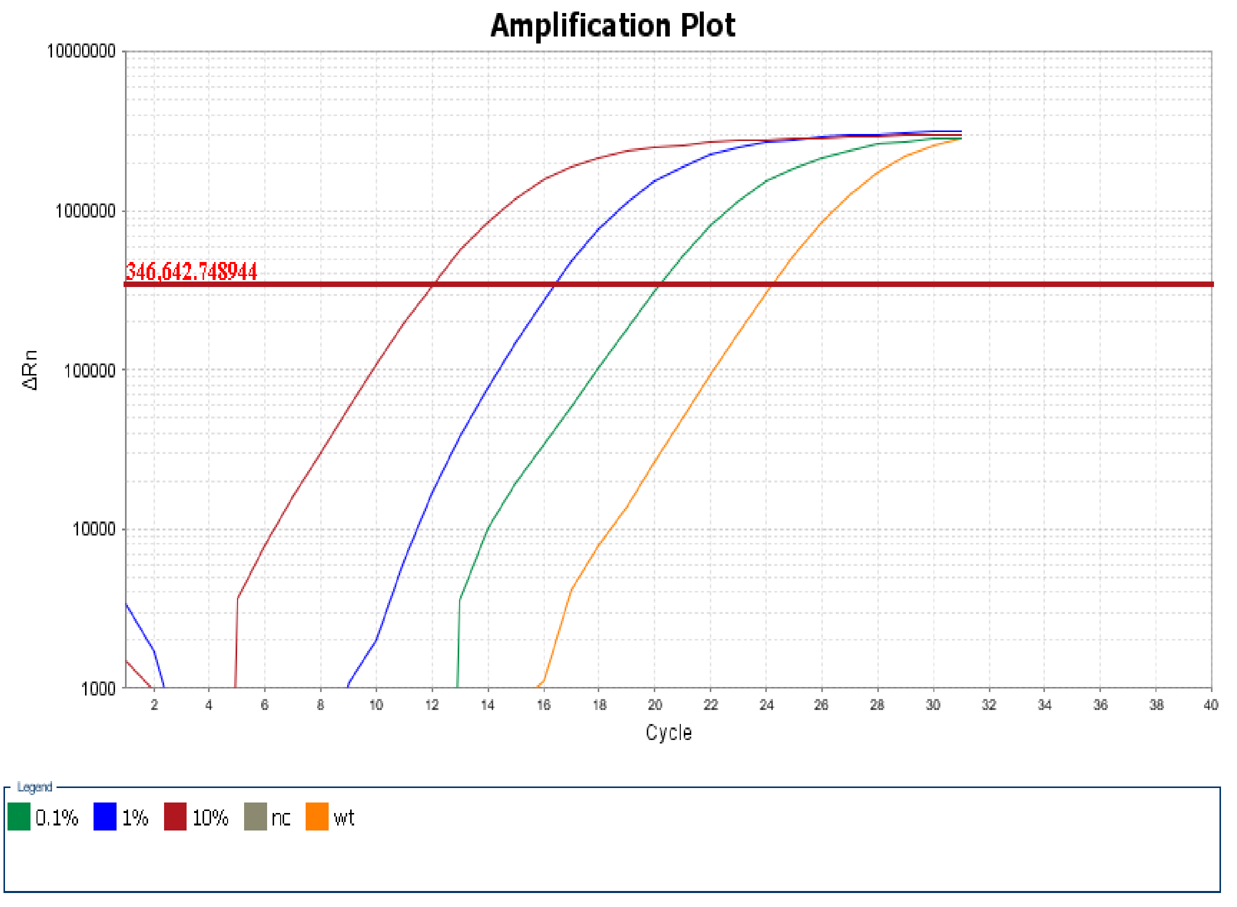

Fig. S26 The amplification plot of fluorescence qPCR detection of *EGFR* gene e19del mutation using a commercial kit. The curves with different colors represent 1e5 copies of plasmid templates with a mutation rate of 10%, 1%, 0.1%, 0 (WT), and NC, respectively.

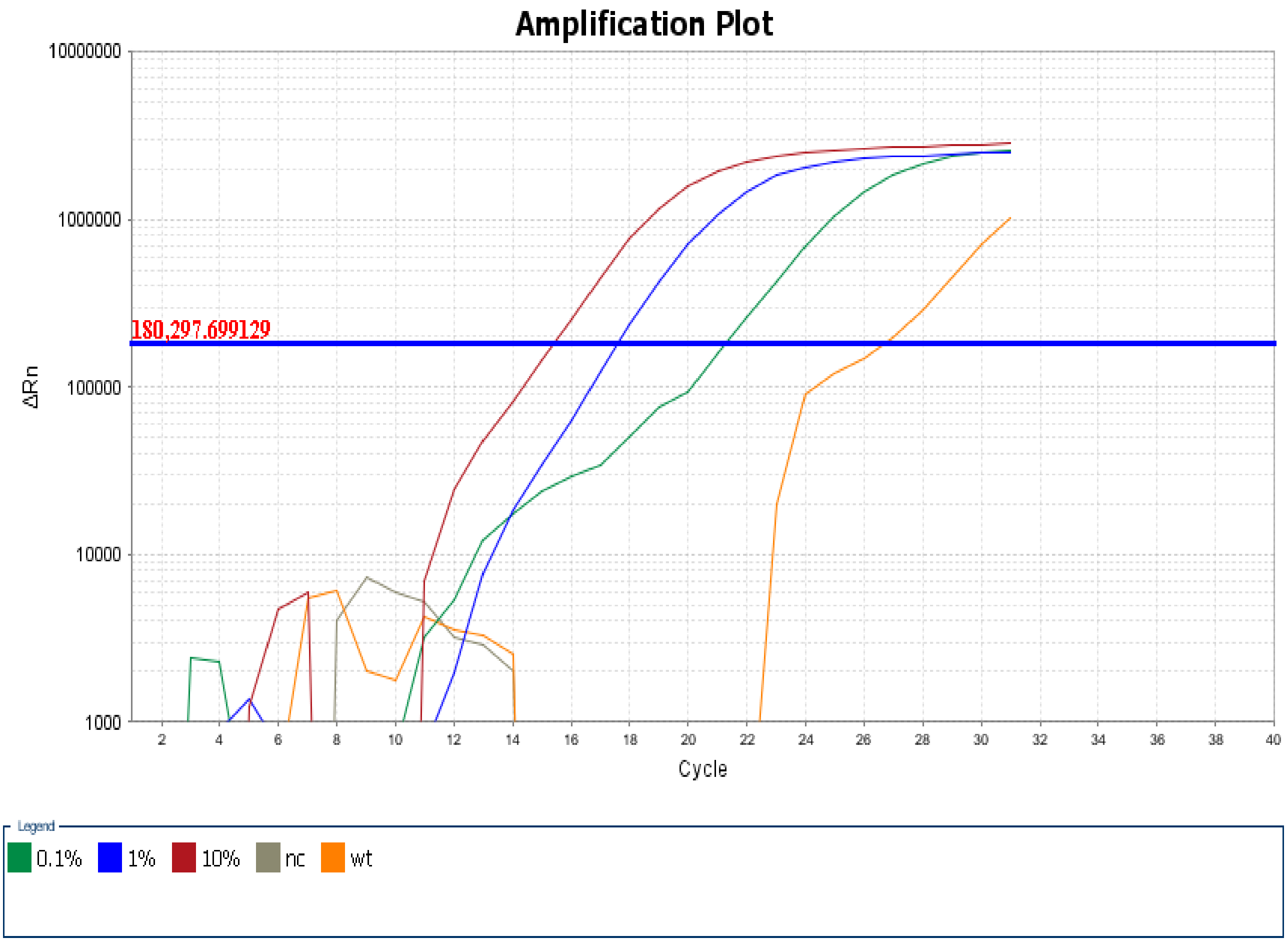

Fig. S27 The amplification plot of fluorescence qPCR detection of *EGFR* gene L858R mutation using a commercial kit. The curves with different colors represent 1e5 copies of plasmid templates with a mutation rate of 10%, 1%, 0.1%, 0 (WT), and NC, respectively.

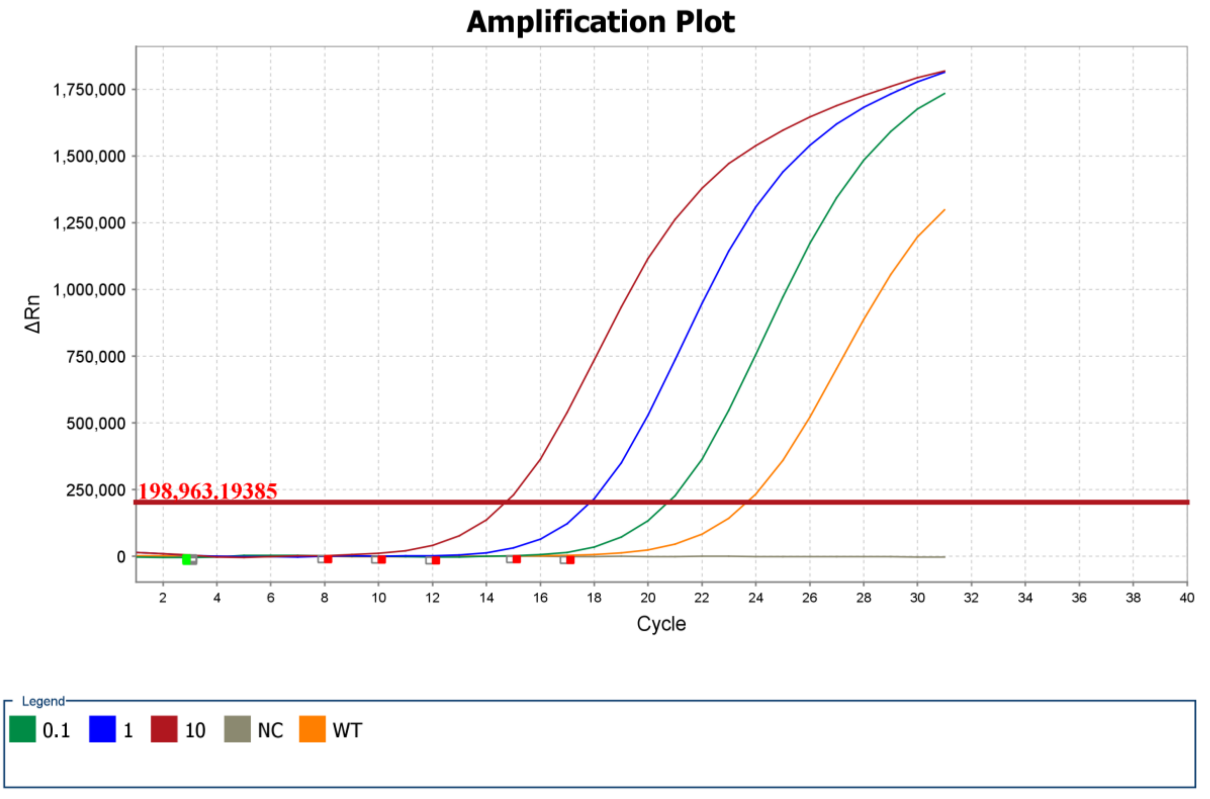

Fig. S28 The amplification plot of fluorescence qPCR detection of *NRAS* gene G12D mutation using a commercial kit. The curves with different colors represent 1e5 copies of plasmid templates with a mutation rate of 10%, 1%, 0.1%, 0 (WT), and NC, respectively.

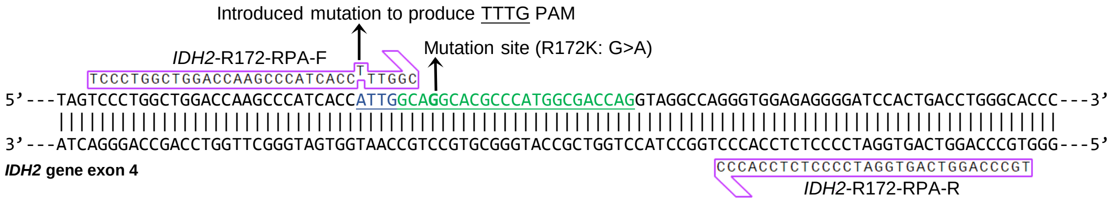

Fig. S29 The RPA primer design for *IDH2*-R172K mutation detection. Forward and reverse primers are framed in purple.
