# Supplementary material for: EasyCatch, a convenient, sensitive and specific CRISPR detection system for cancer gene mutations
Source: Mol Cancer. 2021 Dec 2;20:157. doi: 10.1186/s12943-021-01456-x (PMC8638196; doi:10.1186/s12943-021-01456-x)
Supplement: Supplementary file 4 — Additional file 4: Supplementary Table 1 PCR and RPA primer sequences. Supplementary Table 2 crRNA sequences. Supplementary Table 3 Next-generation sequencing primer sequences. Supplementary Table 4 Primers and probes of Taqman qPCR. Supplementary Table 5 Cas12a nuclease natural and engineered variants. [file 12943_2021_1456_MOESM4_ESM.pdf]

Table S1. PCR and RPA primer sequences

ID	Name	Sequence
P1	*FLT3*-D835-675bp-For	GCCTCTCACTTTTGCTCGGA
P2	*FLT3*-D835-675bp-Rev	AGGATTGCACTCAAAGGCCC
P3	T-vec-D835Y-For	GGCTCGATATATCATGAGTGATTCC
P4	T-vec-D835Y-Rev	CATGATATATCGAGCCAATCCAAAG
P5	T-vec-D835H-For	GGCTCGACATATCATGAGTGATTCC
P6	T-vec-D835H-Rev	CATGATATGTCGAGCCAATCCAAAG
P7	T-vec-D835V-For	GGCTCGAGTTATCATGAGTGATTCC
P8	T-vec-D835V-Rev	CATGATAACTCGAGCCAATCCAAAG
P9	T-vec-D835F-For	GGCTCGATTTATCATGAGTGATTCC
P10	T-vec-D835F-Rev	CATGATAAATCGAGCCAATCCAAAG
P11	*FLT3*-D835-351bp-For	GGTACCTCCTACTGAAGTTG
P12	*FLT3*-D835-351bp-Rev	GTAAGCAGACTGCTGTGAGG
P13	*FLT3*-D835-RPA-F1	AAAGTGGTGAAGATATGTGACTTTGGATTGGC
P14	*FLT3*-D835-RPA-F2	GGTGAAGATATGTGACTTTGGATTGGCTCG
P15	*FLT3*-D835-RPA-F3	CTCCAGGATAATACACATCACAGTAAATAACAC
P16	*FLT3*-D835-RPA-R1	CACAACACAAAATAGCCGTATAAAAATAAGTAGG
P17	*FLT3*-D835-RPA-R2	TTTACCATGATAACGACACAACACAAAATAGCC
P18	*FLT3*-D835-RPA-R3	CCTTTTAAGCATAAGTAAGCAGACTGCTGTGAGGG
P19	*IDH2*-R172-506bp-For	CATGAAGAATTTTAGGACCC
P20	*IDH2*-R172-506bp-Rev	CCAGCCTCACCTCGTCGGTG
P21	T-vec-R172K-For	ATTGGCAAGCACGCCCATGG
P22	T-vec-R172K-Rev	GGCGTGCTTGCCAATGGTGA
P23	*EGFR*-L858-402bp-For	AGCCATAAGTCCTCGACGTG
P24	*EGFR*-L858-402bp-Rev	CTGCGAGCTCACCCAGAATG
P25	T-vec-L858R-For	TTTGGGCGGGCCAAACTGCTGG
P26	T-vec-L858R-Rev	TTTGGCCCGCCCAAAATCTGTGATC
P27	*NRAS*-G12-299bp-For	AATGGAAGGTCACACTAGGG
P28	*NRAS*-G12-299bp-Rev	ACAGAATATGGGTAAAGATG
P29	T-vec-G12D-For	GGAGCAGATGGTGTTGGGAA
P30	T-vec-G12D-Rev	CAACACCATCTGCTCCAACC
P31	*IDH2*-R172-RPA-F	TCCCTGGCTGGACCAAGCCCATCACCTTTGGC
P32	*IDH2*-R172-RPA-R	TGCCCAGGTCAGTGGATCCCCTCTCCACCC
P33	*EGFR*-e19-RPA-F	TCCCAGAAGGTGAGAAAGTTCAAATTCCCGTCGC
P34	*EGFR*-e19-RPA-R	TTCAGAGCCATGGACCCCCACACAGCAAAGC
P35	*EGFR*-L858-RPA-F	AACGTACTGGTGAAAACACCGCAGCATGTC
P36	*EGFR*-L858-RPA-R	CACCTCCTTACTTTGCCTCCTTCTGCATGG
P37	*NRAS*-G12-RPA-F	TTCTTGCTGGTGTGAAATGACTGAGTACAAACTG
P38	*NRAS*-G12-RPA-R	TCTATGGTGGGATCATATTCATCTACAAAGTGG

* Mutated bases are colored in red. The brown base T in primer P31 is an introduced mutation to form a TTTG PAM for the detection of *IDH2*-R172K. The brown base C in primer P33 is an introcuced mutation to destroy an unwanted SaqAI restriction site near the target *EGFR*-e19del site.

Table S2. crRNA sequences

Name	Sequence
FLT3-D835Y-crRNA1	UAAUUUCUACUAAGUGUAGAUGAUUGGCUCGAUAUAUCAUGAGU
FLT3-D835Y-crRNA2	UAAUUUCUACUAAGUGUAGAUGAUUGGCUCGAUACAUCAUGAGU
FLT3-D835Y-crRNA3	UAAUUUCUACUAAGUGUAGAUGAUUGGCUCUAUAUAUCAUGAGU
FLT3-D835Y-crRNA4	UAAUUUCUACUAAGUGUAGAUGAUUGGCUCGAUAUAUAAUGAGU
FLT3-D835H-crRNA1	UAAUUUCUACUAAGUGUAGAUGAUUGGCUCGACAUAUCAUGAGU
FLT3-D835H-crRNA2	UAAUUUCUACUAAGUGUAGAUGAUUGGCUCGACACAUCAUGAGU
FLT3-D835V-crRNA1	UAAUUUCUACUAAGUGUAGAUGAUUGGCUCGAGUUAUCAUGAGU
FLT3-D835V-crRNA2	UAAUUUCUACUAAGUGUAGAUGAUUGGCUCGCGUUAUCAUGAGU
FLT3-D835V-crRNA3	UAAUUUCUACUAAGUGUAGAUGAUUGGCUCGAGUUCUCAUGAGU
FLT3-D835F-crRNA	UAAUUUCUACUAAGUGUAGAUGAUUGGCUCGAUUCAUCAUGAGU
FLT3-D835WT-crRNA1	UAAUUUCUACUAAGUGUAGAUGAUUGGCUCGAGAUAUCAUGAGU
FLT3-D835WT-crRNA2	UAAUUUCUACUAAGUGUAGAUGAUUGGCUCGAGACAUCAUGAGU
FLT3-D835WT-crRNA3	UAAUUUCUACUAAGUGUAGAUGAUUGGCUCUAGAUAUCAUGAGU
FLT3-D835WT-crRNA4	UAAUUUCUACUAAGUGUAGAUGAUUGGCUCGAGAUAUAAUGAGU
IDH2-172WT-crRNA	UAAUUUCUACUAAGUGUAGAUGCAGGCGCGCCCAUGGCGACCAG
IDH2-R172K-CRRNA	UAAUUUCUACUAAGUGUAGAUGCAAGCGCGCCCAUGGCGACCAG
EGFR-e19WT-crRNA	UAAUUUCUACUAAGUGUAGAUGGAGAUGUUGCUUCUCUUAAUUC
EGFR-e19del-crRNA	UAAUUUCUACUAAGUGUAGAUGGAGAUGUUUUGAUAGCGACGGG
EGFR-858WT-crRNA	UAAUUUCUACUAAGUGUAGAUGGCUGGCUAAACUGCUGGGUGCG
EGFR-L858R-crRNA	UAAUUUCUACUAAGUGUAGAUGGCGGGCUAAACUGCUGGGUGCG
NRAS-12WT-crRNA	UAAUUUCUACUAAGUGUAGAUCCAACACCACCCGCUCCAACCAC
NRAS-G12D-crRNA	UAAUUUCUACUAAGUGUAGAUCCAACACCAUCCACUCCAACCAC

* Target sequences, mutated bases, and introduced mismatches are colored in blue, red and orange, respectively.

Table S3 Next-generation sequencing primer sequences

Name	Sequence
D835-DSF1	ATCACGTCACCGGTACCTCCTACTGA
D835-DSF2	CGATGTTCACCGGTACCTCCTACTGA
D835-DSF3	TTAGGCAGTCACCGGTACCTCCTACTGA
D835-DSF4	TGACCAGTCACCGGTACCTCCTACTGA
D835-DSF5	ACAGTGCTTCACCGGTACCTCCTACTGA
D835-DSF6	GCCAATTCACCGGTACCTCCTACTGA
D835-DSF7	CAGATCTTCACCGGTACCTCCTACTGA
D835-DSF8	ACTTGAAATCACCGGTACCTCCTACTGA
D835-DSF9	GATCAGGTCACCGGTACCTCCTACTGA
D835-DSF10	TAGCTTCCTCACCGGTACCTCCTACTGA
D835-DSF11	GGCTACTCACCGGTACCTCCTACTGA
D835-DSF12	CTTGTATCACCGGTACCTCCTACTGA
D835-DSF13	AGTCAATCACCGGTACCTCCTACTGA
D835-DSF14	AGTTCCTTCACCGGTACCTCCTACTGA
D835-DSF15	ATGTCAGCTCACCGGTACCTCCTACTGA
D835-DSF16	CCGTCCATTCACCGGTACCTCCTACTGA
D835-DSF17	GTAGAGCTCACCGGTACCTCCTACTGA
D835-DSF18	GTCCGCATCACCGGTACCTCCTACTGA
D835-DSF19	GTGAAATCTCACCGGTACCTCCTACTGA
D835-DSF20	GTGGCCTCACCGGTACCTCCTACTGA
D835-DSF21	GTTTCGTCACCGGTACCTCCTACTGA
D835-DSF22	CGTACGGTCACCGGTACCTCCTACTGA
D835-DSF23	GAGTGGAGTCACCGGTACCTCCTACTGA
D835-DSF24	GGTAGCATCACCGGTACCTCCTACTGA
D835-DSF25	ACTGATGTCACCGGTACCTCCTACTGA
D835-DSF26	ATGAGCCATCACCGGTACCTCCTACTGA
D835-DSF27	ATTCCTTCACCGGTACCTCCTACTGA
D835-DSF28	CACCGGCATCACCGGTACCTCCTACTGA
D835-DFR	GAAATAGCAGCCTCACATTGCC

Table S4 Primers and probes of Taqman qPCR

Name	Sequence
835-qPCR-F	cgggaaagtggtgaagatatgtg
835-qPCR-R	ctgacaacatagttggaatcactcatg
D835Y-probe 1	FAM-ctcgaGatatcatgagtg-MGB
D835Y-probe 2	FAM-ttggattggctcgaGatat-MGB

* D835Y mutated bases were colored in red.

Table S5 Cas12a nuclease natural and engineered variants

Name	Genotype	Size (aa)	PAM (5' to 3')	PMID
LbCas12a	WT	1,228	TTTV	26422227
LbCas12a-RR	G532R, K595R	1,228	TYCV	28581492
LbCas12a-RVR	G532R, K538V, Y542R	1,228	TATV	28581492
LbCas12a-RVRR	G532R, K538V, Y542R, K595R	1,228	TNTN, TACV, TTCV, CTCV, CCCV	32107556
impLbCas12a	D156R, G532R, K538V, Y542R, K595R	1,228	TTTV	32107556
AsCas12a (AsCpf1)	WT	1,308	TTTV	26422227
AsCas12a-RR	S542R, K607R	1,308	TYCV	28581492
AsCas12a-RVR	S542R, K548V, N552R	1,308	TATV	28581492
enAsCas12a	E174R, S542R, K548R	1,308	TTYN, VTTV, TRTV	30742127
enAsCas12a-HF	E174R, S542R, K548R, N282A	1,308	TTYN, VTTV, TRTV	30742127
AsCas12a-Ultra	M537R, F870L	1,308	TTTV	34162850
FnCas12a	WT	1300	TTN	26422227
FnCas12a-RR	N607R, K671R	1300	TTYV, TCCV	30239882
FnCas12a-RVR	N607R, K613V, N617R	1300	TWTV	30239882
MbCas12a	WT	1373	TTN	30190308
MbCas12a-RR	N576R, K637R	1373	TTYV, TCCV	30239882
MbCas12a-RVR	N576R, K582V, N586R	1373	TWTV	30239882
